# Biantennary oligoglycines and glyco-oligoglycines self-associating in aqueous medium

**DOI:** 10.3762/bjoc.10.140

**Published:** 2014-06-17

**Authors:** Svetlana V Tsygankova, Alexander A Chinarev, Alexander B Tuzikov, Nikolai Severin, Alexey A Kalachev, Juergen P Rabe, Alexandra S Gambaryan, Nicolai V Bovin

**Affiliations:** 1 Shemyakin-Ovchinnikov Institute of Bioorganic Chemistry, ul. Miklukho-Maklaya 16/10 Moscow V-437, 117997, Russia; 2 Department of Physics, Humboldt University Berlin, Newtonstr. 15, D-12489, Berlin, Germany; 3 Plasmachem GmbH, Rudower Chaussee 29, D-12489 Berlin, Germany; 4 M. P. Chumakov Institute of Poliomyelitis and Viral Encephalitides, 142782 Moscow Region, Russia

**Keywords:** glycopeptides, influenza virus, multivalent glycosystems, oligoglycine, polyglycine II, self-assembling, tectomers

## Abstract

Oligoglycines designed in a star-like fashion, so-called tri- and tetraantennary molecules, were found to form highly ordered supramers in aqueous medium. The formation of these supramers occurred either spontaneously or due to the assistance of a mica surface. The driving force of the supramer formation is hydrogen bonding, the polypeptide chain conformation is related to the folding of helical polyglycine II (PG II). Tri- and tetraantennary molecules are capable of association if the antenna length reach 7 glycine (Gly) residues. Properties of similar biantennary molecules have not been investigated yet, and we compared their self-aggregating potency with similar tri- and tetraantennary analogs. Here, we synthesized oligoglycines of the general formula R-Gly*_n_*-Х-Gly*_n_*-R (X = -HN-(СН_2_)*_m_*-NH-, *m* = 2, 4, 10; *n* = 1–7) without pendant ligands (R = H) and with two pendant sialoligands (R = sialic acid or sialooligosaccharide). Biantennary oligoglycines formed PG II aggregates, their properties, however, differ from those of the corresponding tri- and tetraantennary oligoglycines. In particular, the tendency to aggregate starts from Gly_4_ motifs instead of Gly_7_. The antiviral activity of end-glycosylated peptides was studied, and all capable of assembling glycopeptides demonstrated an antiviral potency which was up to 50 times higher than the activity of peptide-free glycans.

## Introduction

Recently, we have synthesized and described tetraantennary [2] and triantennary [3] oligoglycines capable of spontaneous or surface-promoted formation of flat layers in aqueous medium. These layers are one or two molecules thick. The stability was attributed to the formation of a network of hydrogen bonds. This class of supramers has been called tectomers. Tectomers in a layer are packed by polyglycine II type (PG II) [4–5]. Their helical polypeptide chain fundamentally differs from the canonical α-helix. The association of symmetrical tetra- and triantennary (star-like) oligoglycines spontaneously proceeds only when the number of glycine (Gly) residues in a chain (*n*) is equal or greater to seven. Oligoglycines with an antennae size less than seven do either not associate at all or require extremely favorable conditions, in particular surface promotion. Yet, the properties of similar biantennary molecules were not investigated. Here, we synthesized biantennary oligoglycines and studied them in order to determine the necessary and sufficient conditions for self-association. More specifically, we investigated the combination of structure elements, such as the *n* value, the type of terminal substituents, and the type of structural motifs (core), where the antennae are connected to each other. The knowledge of the rules found for the unsubstituted assembly of oligoglycines may be suitable for us for the design of corresponding sialo derivartives, which are candidate therapeutics for the blocking of the influenza virus [6].

## Results and Discussion

### Synthesis of biantennary oligoglycines and their glyco derivatives

**Figure 1:**
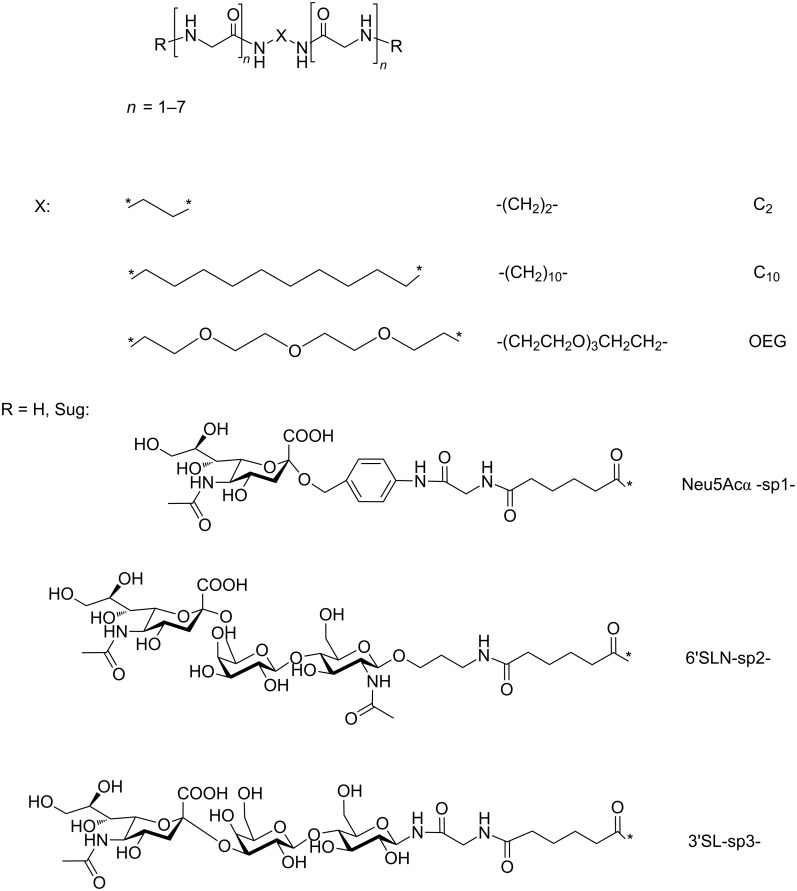
The structure of biantennary oligoglycines and their glyco derivatives (sp = spacer group).

The synthesized biantennary oligoglycines and their glyco derivatives are presented in Figure 1. Analogously to tri- and tetraantennary molecules, oligoglycine antennas are connected according to the ‘head-to-head’ principle, i.e., by their C-termini, so that the two amino groups are terminal. The obtained compounds differ threefold. Firstly, they differ by core X nature: hydrophilic oligoethylene glycol (OEG), hydrophobic flexible decamethylene (C_10_), or short ethylene (C_2_). Secondly, the length of oligoglycine antennas, i.e., the number of glycine residues in a chain (*n* = 1–7) is different. Thirdly, the substances differ by the presence or absence of carbohydrate fragment (Sug), containing α-*N*-acetylneuraminic moiety (Neu5Acα).

**Scheme 1:**
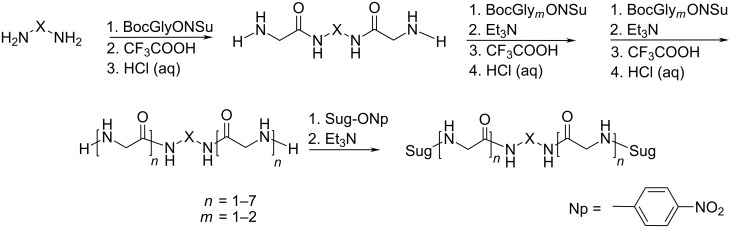
Synthesis of biantennary oligoglycines and their glycoderivatives.

Diamines NH_2_–X–NH_2_ were the starting substances for the synthesis, oligoethylene glycol diamine was obtained from ditosylate as described in [7–8]. The synthesis of biantennary oligoglycines was carried out by means of the activated esters method (Scheme 1) [9]. The glycine chains were elongated stepwise by their N-terminus by using *N*-oxysuccinimidyl esters (BocGlyONSu or BocGly_2_ONSu, Boc = *tert*-butyloxycarbonyl). Boc-peptides were isolated from the reaction mixture by the removal of the solvent and the re-crystallization of the reaction product from aqueous methanol (yields 75–95%). In the case of poorly soluble products the impurities and starting materials were washed off with methanol (yields 60–90%). In the case of an oil-like substance (Х = ОEG, *n* = 2) chromatography on silica gel was performed. The quantitative removal of Boc groups was achieved by the treatment of the obtained peptides with trifluoroacetic acid. Salt forms (trifluoroacetates or hydrochlorides) of diamino derivatives were obtained by sedimentation from an aqueous solution by methanol (yield ≥95%). At later stages of elongation the salts were converted to the respective free bases by treatment with a slight excess of triethylamine. The preparation of oligoglycines with a chain length exceeding five glycine residues for the derivatives with core C_10_ and six residues for core C_2_ failed due to their low solubility and, consequently, the impossibility of separating them from the intermediates of the synthesis.

Sialo conjugates of biantennary oligoglycines were obtained from the corresponding diamines and derivatives of α-*N*-acetylneuraminic acid (Sug-ONp), where the carboxyl group of the spacer was activated with 4-nitrophenol (Np) (Scheme 1). The synthesis of these compounds was described in [10–11]. Owing to the poor solubility of the diamine form of oligoglycines with cores C_2_ and C_10_ in DMSO, the reaction was carried out in a saturated aqueous solution of lithium bromide, which prevented the formation of hydrogen bonds and thus increased the solubility of oligoglycines. Glycopeptides were isolated from the reaction mixture by gel-permeation chromatography (yields 70–75%). The peptide modification by the amino group with mono- or oligosaccharides dramatically increased their solubility in water. This may support their antiviral action (see below), because glycopeptides act topically, in the respiratory tract, and are administered as a spray.

We then investigated the ability of synthesized biantennary oligoglycines to assemble in aqueous media as well as the antiviral activity of glycoderivatives.

### Study of biantennary oligoglycines association in solution by dynamic light scattering

The size of the particles formed by the biantennary oligoglycines in solution was measured with the dynamic light scattering method (DLS). We found that the ability of association depends on the number of the glycine units in the antennae, the nature of the core, the pH, and the peptide concentration.

It is known that the charge of terminal amino groups of the protonated form of oligoglycines hinders association. To overcome this obstacle an equimolar quantity of NaHCO_3_ or Nа_2_CO_3_ was added to aqueous solutions of oligoglycine salts. In the absence of the base, pH values of oligoglycine salt solutions varied from 3.5 to 4.5 (hereinafter denoted as рН < 5). In the case of the addition of one base equivalent per one amino group the solution becomes neutral (рН 6.5), in the case of two Nа_2_CO_3_ equivalents the pH value is more than 8.5 (basic solution, denoted as pH > 8.5).

At *n* < 4 peptides with an oligoethylene glycol core and the cores С_2_ and С_10_ did not form associates in aqueous medium in all the studied ranges of pH and concentration (0.1–1.0 mg/mL).

Biantennary oligoglycines, cores С_2_ and С_10,_ *n* ≥ 4, are capable of forming associates (700–900 nm) in acidic solutions in the studied concentration range, except for Н-Gly_4_NH(СН_2_)_10_NHGly_4_-Н·2HCl, which associates in concentrations ≥0.5 mg/mL.

**Figure 2:**
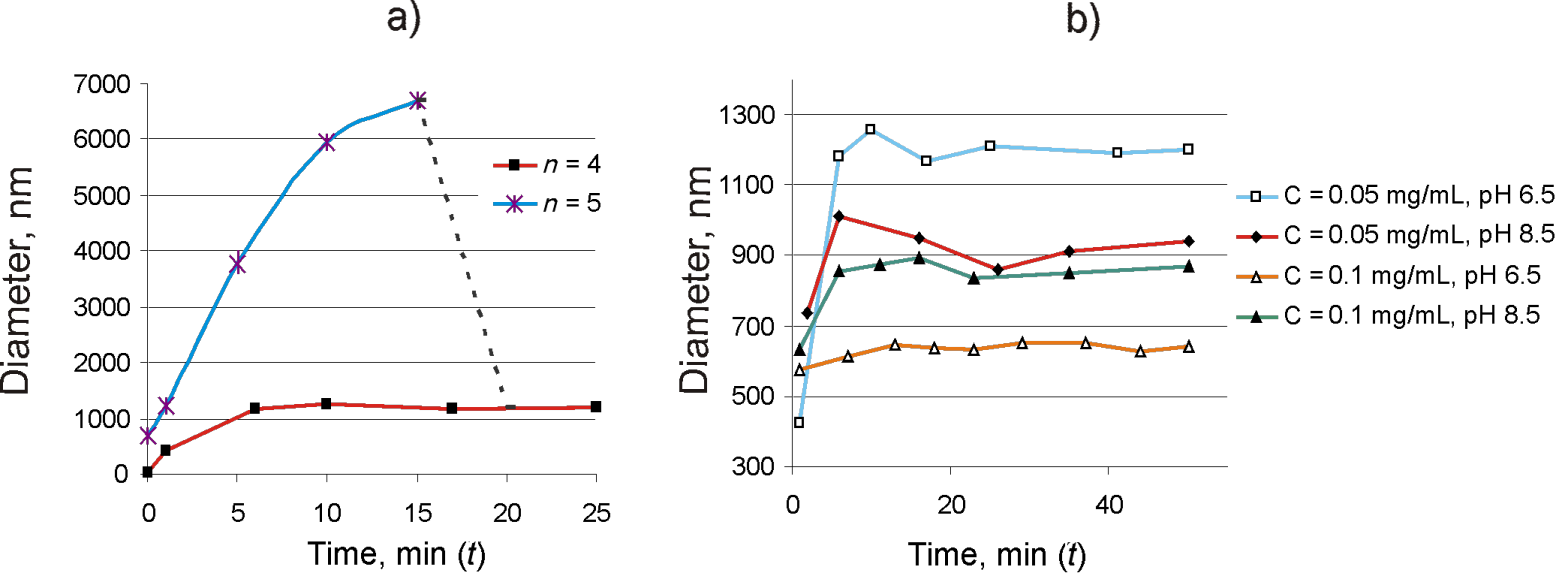
Dynamics of associate formation by biantennary oligoglycines Н-Gly*_n_*-NH(СН_2_)_10_NH-Gly*_n_*-Н. a) *n* = 4–5, in aqueous solution at рН 6.5 and a concentration of 0.1 mg/mL at *t* = 0 and рН < 5 before the addition of base and at *t* > 0 and рН 6.5 after the addition of base. The region of sedimentation is marked with a dotted line. b) *n* = 4, in aqueous solution at рН 6.5 and 8.5.

Molecules with the core С_2_ (*n* = 4–6) and C_10_ (*n* = 5) associate so rapidly in neutral and basic media that a precipitate is formed (data for peptide with *n* = 5 are given in Figure 2a). Only the peptide Н-Gly_4_NH(СН_2_)_10_NHGly_4_-Н in concentration ≤0.1 mg/mL is capable of forming associates (800–1200 nm) stable in aqueous media (Figure 2a,b).

### Study of biantennary peptides association using scanning force microscopy

Scanning force microscopy (SFM) elucidates information not only about the association process both in solution and on a surface, but also about fine details of the formed architectures. Of particular interest are cases characterized by the active participation of the surface in accelerating the self-assembly. To discriminate the processes taking place on the surface from similar processes in liquid volume, measurements were carried out immediately after the deprotonation of oligoglycine salts at incubation times insufficient for a spontaneous association in solution (found out as ≤1 min). The solution was placed on a freshly cleaved surface of mica or graphite, exposed for fixed time intervals (denoted as *t*_exp_), followed by the removal of the liquid phase from the surface and the scanning of the sample in tapping mode in air. The contact mode of scanning was used for experiments in a liquid cell. Experiments in a liquid cell allowed us to study the kinetics of the process without a possible distortion of the nanostructures resulting from the drying of the sample.

**Figure 3:**
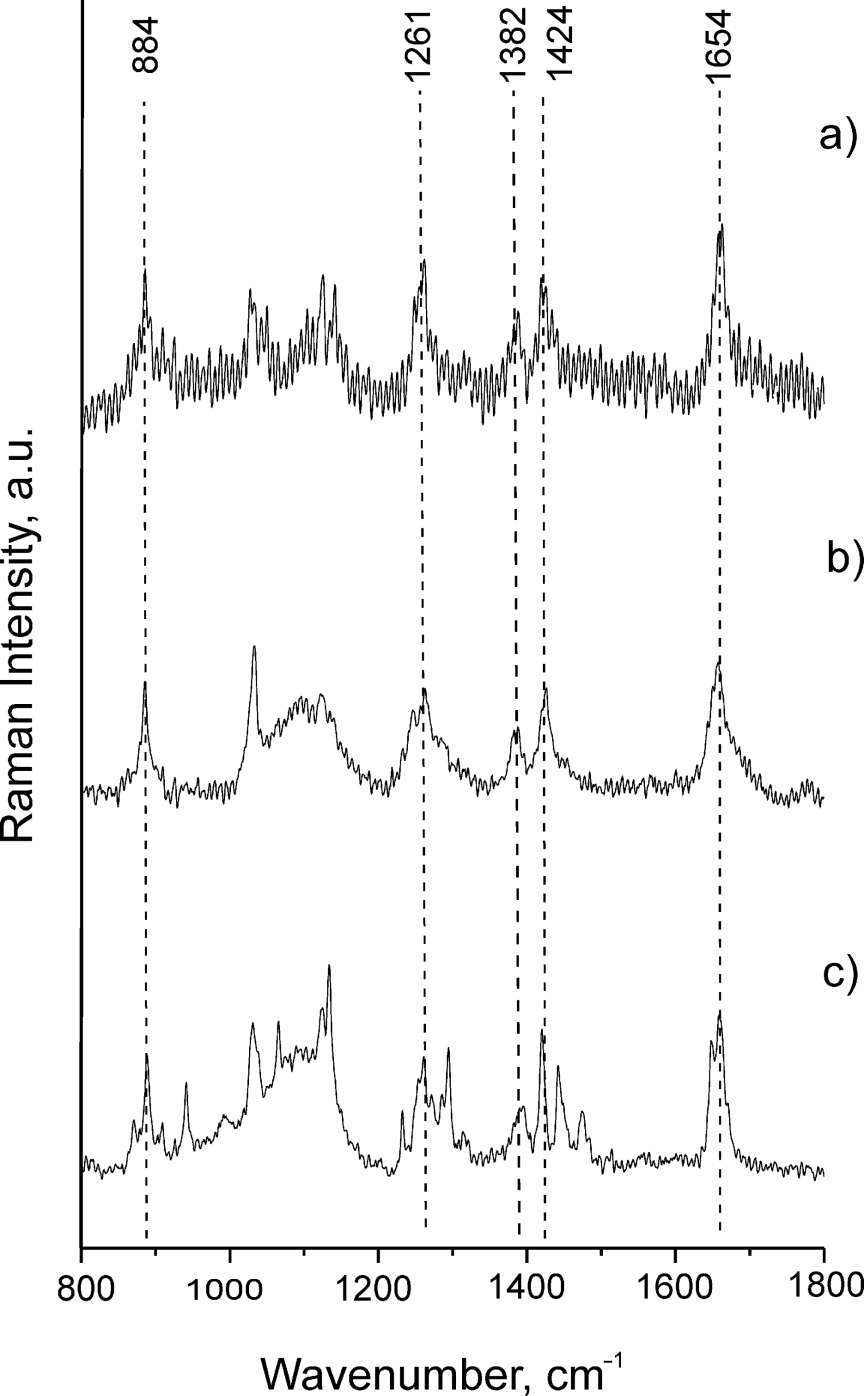
Raman spectra of a) [H-Gly_7_-NHCH_2_]_4_C; b) H-Gly_4_-NH(CH_2_)_2_NH-Gly_4_-H; c) H-Gly_4_-NH(CH_2_)_10_NH-Gly_4_-H. The spectra contain characteristic bands at 884, 1261, 1382, 1424 and 1654 cm^−1^, corresponding to the structure PG II [12]. Spectra were recorded for the samples in solid phase.

The Raman spectra (Figure 3) of biantennary oligoglycines capable of association as well as the spectra of tri- and tetraantennary peptides described earlier display bands at 884, 1261, 1382, 1424 and 1654 cm^−1^, which are characteristic and specific for crystalline PG II. Based on the presence of these bands we conclude that the structure organization of associates formed in solution corresponds to PG II. The sensitivity of routine Raman scattering method is insufficient for the work with oligoglycine monolayers, so indirect methods were used in order to attribute formed material to a PG II structure. More specifically, geometrical parameters of the layers were determined by using SFM and compared with: 1) those for tectomers (attributed to PG II, see above) formed in solution and 2) calculated values for different, not only PG II, models. As shown below for particular examples, in most cases spontaneous and surface-mediated assembly led to associates of PG II structure, i.e., tectomers.

**Figure 4:**
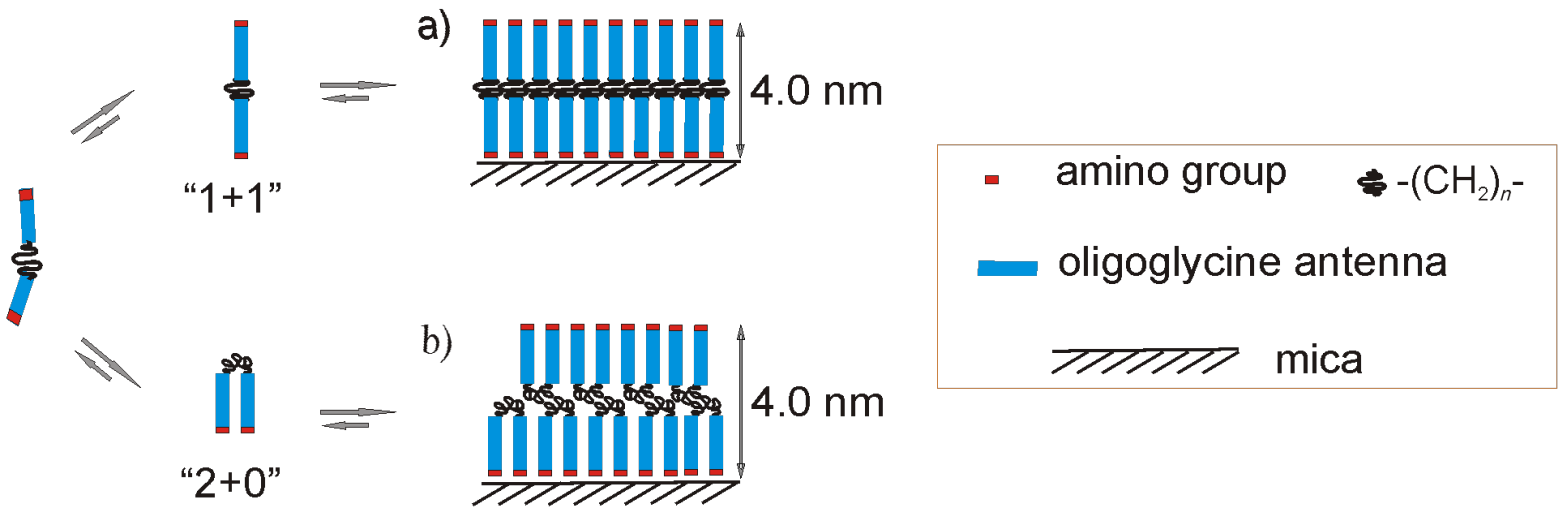
Model of the formation of tectomer layers by biantennary oligoglycines on a mica surface. The heights are given for Н-Gly_4_NH(СН_2_)_10_NHGly_4_-Н.

The formation of the PG II structure for oligoglycines with a short rigid spacer С_2_ is only possible if the molecule is extended, i.e., antennas are pointing in opposite directions (conformation “1 + 1”, Figure 4а). The presence of a flexible core (C_10_, OEG) allows the molecule to adopt the conformation ”2 + 0”, which is characterized by unidirectional oligoglycine antennas. The hydrophobic side of the tectomer should initiate the formation of the second layer with the opposite orientation of the monomer in aqueous solutions (Figure 4b) in order to minimize the thermodynamically unfavorable contact with water.

It was demonstrated for biantennary oligoglycines that a concentration of 0.1 mg/mL is optimal for the study of the dynamics of tectomer growth on a mica surface. At higher concentrations the growth both in solution and on the surface proceeded so rapidly that the dynamics study was considered impossible. No tectomer structure was observed under acidic conditions (рН < 5), whereas under neutral and basic conditions the reaction proceeded similarly in terms of both the velocity and the morphology of formed tectomers. The oligoethylene glycol derivatives Н-Gly*_n_*-NH(СН_2_СН_2_О)_3_СН_2_СН_2_NH-Gly*_n_*-Н (*n* = 2–7), non-associating in aqueous solutions as well as oligoglycines with cores С_2_ and С_10_ (*n* < 4) did not form associates on a mica surface under all studied ranges of pH (from 4.5 to 8.5).

**Figure 5:**
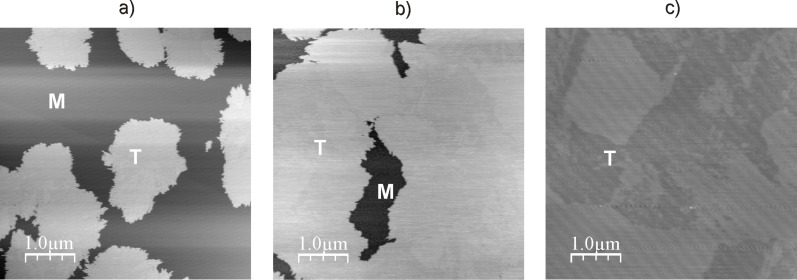
Growth of the tectomer formed by the peptide Н-Gly_4_-NH(СН_2_)_10_-NHGly_4_-Н (concentration 0.1 mg/mL) on a mica surface at рН 6.5, with tapping mode, SFM on air, and a *t*_еxp_ of а) 0.5 min, b) 1 min, c) 2 min. Here, the shown field is completely covered with tectomer layer; its roughness found to be ± 0.1 nm. Т indicates the tectomer layer, M the uncovered mica regions.

According to dynamic light scattering data (see above) only peptide Н-Gly_4_NH(СН_2_)_10_NHGly_4_-Н was capable of forming tectomers in neutral and basic solutions which were unchanged in an aqueous phase for a long time. Figure 5 demonstrates the dynamics of layer growth on mica with the characteristic formation of islet structures (*t*_exp_ = 0.5 min, Figure 5а), growing laterally (*t*_exp_ = 1 min, Figure 5b), and covering the whole surface with an even layer (*t*_exp_ = 2 min, Figure 5c). Presumably, longer times (*t*_exp_ > 2 min) are characterized by the appearance of multilayer tectomers resulting from the sorption of associates formed in solution. The multilayer tectomers can be readily removed by washing with buffer solution (рН 6.5 or 9.0). The morphology of the first layer remains unchanged and the available defects (‘holes’) are preserved. The layer height is 3.7–4.0 nm, which may correspond to both mono- and bilayer (conformations “1 + 1” and “2 + 0”, respectively, see Figure 4).

**Figure 6:**
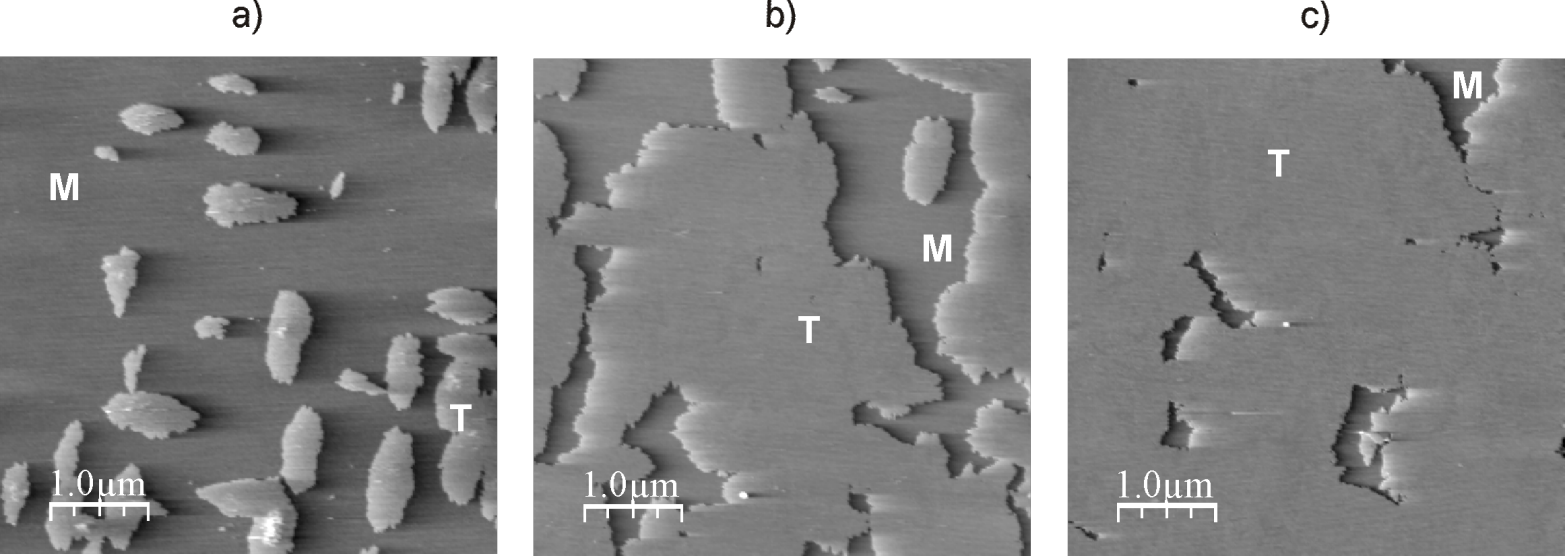
Growth of the tectomer formed by peptide Н-Gly_4_-NH(СН_2_)_10_NH-Gly_4_-Н (concentration 0.1 mg/mL) on a mica surface in a liquid cell at рН 6.5. Phase SFM images were taken with a) *t*_exp_ = 1 min, b) *t*_exp_ = 2 min and c) *t*_exp_ = 3 min where *t*_exp_ is the time after the experiment was started. Т indicates the tectomer layer, M the uncovered mica regions.

The dynamics of Н-Gly_4_NH(СН_2_)_10_NHGly_4_-Н association was studied in more detail in a liquid cell (Figure 6). After 3 min the surface was virtually completely covered with a uniform defect-free layer. It should be noted that the stepwise surface profile (typical for bilayer structures) was not observed in a liquid cell. The layer morphology was identical to the one observed in experiments in air (Figure 5).

**Figure 7:**
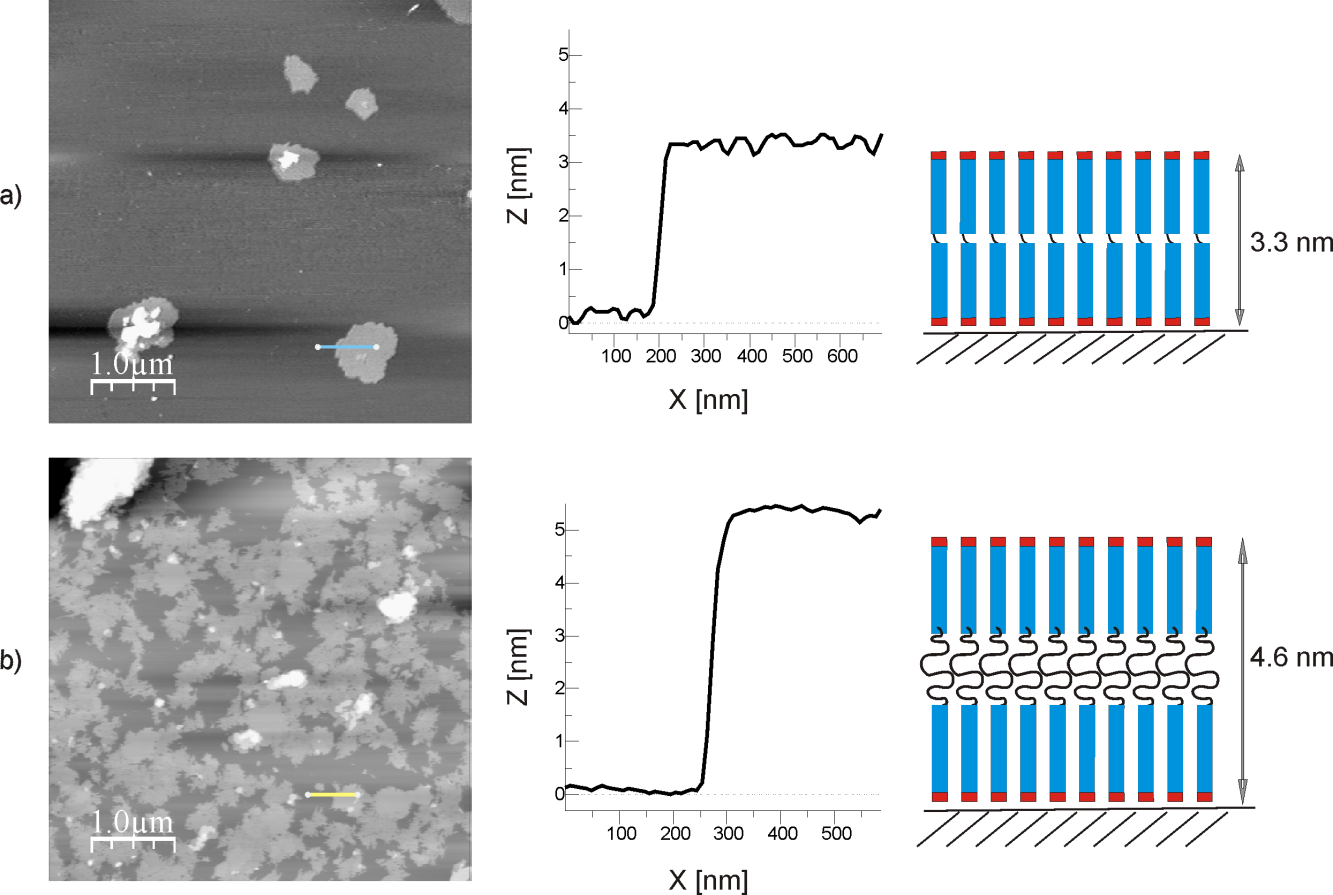
SFM images of associates formed by peptides а) Н-Gly_5_-NH(СН_2_)_2_NH-Gly_5_-Н and b) Н-Gly_5_-NH(СН_2_)_10_NH-Gly_5_-Н (concentration 0.1 mg/mL) on a mica surface at рН 6.5 with *t*_еxp_ = 10 min SFM tapping mode in air, and an incubation time in solution of 1 min. Surface profiles, schematic layer models, and their calculated heights are given on the right.

Dynamic light scattering data give evidence that in neutral aqueous solutions the association of compounds Н-Gly_5_-NH-X-NH-Gly_5_-Н (cores С_2_ and С_10_) leads to the formation of large aggregates. By means of SFM it was demonstrated that the peptide with core C_2_ formed islet-like tectomers on mica surface (*t*_exp_ = 10 min) with a height of 3.3 nm and planar dimensions of 500–700 nm (Figure 7a). The compound with core C_10_ associated more rapidly (Figure 7b), though the surface was not completely covered (*t*_exp_ = 10 min). This is in contrast to the structure analog with four glycines in the antenna, where a time period of only two minutes was sufficient for complete covering. The measured heights fit the model “1 + 1”.

The obtained data give evidence that mica promotes the formation of tectomers from biantennary oligoglycines in neutral and basic solutions. Layer growth proceeds due to the surface co-participation. In the case of the molecule Н-Gly_4_-NH(СН_2_)_10_NH-Gly_4_-Н growth continues until the surface is completely covered, whereas in the bulk of the liquid phase dimensions remain unchanged over time according to dynamic light scattering data.

There are no direct data pointing at the particular conformation (“1 + 1” or ”2 + 0”) the peptide monomer has in the layer (Figure 4). The height value does not allow for the unambiguous assignment of one of the proposed models. Nevertheless, all intrinsic data supports the model ”1 + 1”: 1) the steps typical for a bilayer profile are not present in the SFM images, 2) intense washing does not lead to the formation of half-height structures, 3) the lack of any association on a graphite surface where the formation of ”2 + 0” is expected to be preferable (see below).

Mica promotes the assembling of amino-terminated chains due to the negative charge of the surface. In contrast, graphite did not participate in the association of biantennary oligoglycines, we observed only irregular associates formed in solution. This experimental result is unexpected, because the formation of a monolayer with the monomer conformation ”2 + 0” is favorable according to molecular dynamics simulations [13].

### Minimal size of Gly*_n_* fragment providing association

In the case of biantennary molecules association formally starts from the value *n* = 4, but, in fact, this value is supposedly equal to 8 because biantennary peptides form a polyglycine structure in the extended conformation ”1 + 1”. It is noteworthy that related polymers, nylons with the formula -NH(CH_2_)_x_CO- are known to form a PG II structure [14], i.e., additional methylene groups (-(CH_2_)_x_- instead of -CH_2_-) have no significant influence on its ability to form PG II. The association of tri- and tetraantennary peptides leads to structures with the monomer conformations ”2 + 2” and ”3 + 0”, respectively. The association starts from *n* = 7. Presumably, the first and closest to forking Gly residue takes a distorted conformation and does not take part in the formation of hydrogen bonds with neighboring residues. In the case of tetraantennary oligoglycines the plane of one pair of antennas is rotated by 90° [8] with respect to another pair, so that the glycines cannot form a continuous chain. On the other hand, in the biantennary analog the chain Gly_4_-Х-Gly_4_ has the ability to form a PG II structure despite the core fragment -(CH_2_)*_n_*-.

### The nature of the core fragment Х in H-Gly*_n_*-Х-Gly*_n_*-H

Biantennary molecules with core С_10_ form tectomers on a mica surface more readily than the molecules with core C_2_ and an equal number of glycines. The more flexible core C_10_ should lead to a entropy driven destabilization. The opposite effect observed in reality is most probably caused by van der Waals interactions of hydrophobic fragments C_10_ closely situated in the PG II structure. The oligoethylene glycol core abolishes the formation of a PG II structure, presumably due to competitive hydrogen bonding with spatially close oligoglycine fragments.

### Spontaneous and surface-promoted association

The formation of tectomers on mica proceeds considerably more rapidly than association in solution (the formation of associates in solutions just starts when assembling on the surface is already finished), i.e., the mica surface obviously plays an active role in the process. Tectomer growth starts from the formation of islet structures that increase in lateral direction and cover the whole surface in an even layer. This growth is limited only by the dimensions of the support itself. It should be noted that graphite, in contrast to mica, does not promote association.

#### Effect of рН value

Depending on the pH value, the free terminal group of the oligoglycine chain can be heavily charged, weakly charged, or neutral. In acidic solutions antennas are repulsed due to the positive charge, which hinders tectomer assembly or even abolishes it. The pH value effects not only the ability to assemble but also the morphology of forming supramers. Thus, in neutral solutions biantennary oligoglycines form multilayer tectomers. The process is unstoppable at the stage of the monolayer formation. At the same time, monolayer tectomers are exclusively formed in basic solutions.

#### Concentration range

Most parts of the experiments were carried out in the concentration range of 0.1–1.0 mg/mL. A concentration of 0.1 mg/mL was used for the adequate comparison of association of all investigated peptides. The association in the liquid phase proceeds slower at low concentrations leading to an increased size of the formed supramers.

In summary, based on our investigations related to unglycosylated molecules we can conclude that the association of biantennary oligoglycines is affected by several factors. 1) Mica but not graphite promotes the formation of tectomers. 2) The spatial organization of oligoglycine molecules in supramers corresponds to PG II conformation. 3) Not less than four glycine residues in each of two antennas are required for the assembling of monomer layers into surface tectomer layers or into long-living associates in solution. 4) Oligoethylene glycol core ‘inhibits’ the association both in the liquid phase and on a mica surface.

#### Antiviral activity of glycoderivatives

The idea of antiadhesion influenza virus therapy is based on the inhibition or the blocking of the binding of the influenza virus with target cells [15]. Monovalent oligosaccharides are incapable of an efficient competition for analogous glycans on the cell surface due to the low binding constant with viral hemagglutinin. An attractive way of increasing the affinity of a blocker (inhibitor) is the design of multivalent receptor analogs such as the oligoglycine-based tectomers described above. A first success for an application in this regard was achieved by inhibiting the influenza virus by sialo derivatives of the associating tetraantennary peptides, which demonstrated an antiviral activity three orders of magnitude higher than the activity of non-associating analogs [2]. Similar triantennary molecules with sialo-glycan located in the molecule “head”, however, appear to display a low activity [16]. Thus, it was interesting to study the antiviral activity of sialo derivatives of biantennary oligoglycines in relation to their propensity to associate in aqueous solutions.

The fact that a sialylated biantennary peptide is capable of association in an aqueous solution similarly to glycan-free peptides is confirmed by DLC data. The average size of the sialoglycopeptide aggregates in aqueous solution was about 1 μm (data not shown).

The antiviral activity of biantennary glycopeptides was studied by means of a fetuin binding inhibition test (FBI-test) [17] (the glycoprotein fetuin contains several sialylated carbohydrate chains). In this test, the glycopeptides inhibited the binding of a fetuin peroxidase conjugate to a virus immobilized on a plastic (related to the corresponding monomer). Results are given in Table 1. The activity of associating sialooligoglycines with core C_2_ was only 3–6 times higher than the activities of their non-associated counterparts (*n* = 2–4) and the monomeric reference sialoside, Neu5AcαBn. The compound with core С_10_ and *n* = 4 demonstrated the highest activity from the studied biantennary glycopeptides, which was 50 times higher than the activity of the monomer. The activity of bivalent derivatives with core OEG and *n* = 2–5 did not exceed that of monovalent sialoside. However, the activity increased dramatically when *n* = 6 (up to 50 times), although it was still orders of magnitude smaller compared to the high activity of polymeric inhibitors [18]. As sialooligoglycines of the OEG series did not associate in aqueous solution, we suppose that the reason for the increased activity is related to a critical distance, which facilitates the realization of a divalent interaction of this bivalent molecule with a viral hemagglutinin. Indeed, a simple calculation demonstrates that this distance in a maximally extended molecule with *n* = 6 is about 100 Å. This distance value corresponds to the distance between the carbohydrate binding sites in one molecule of a hemagglutinin homotrimer and slightly exceeds the distance between a couple of hemagglutinin trimmers, which are closely situated on the virion surface.

**Table 1:** Relative activity of biantennary glycopeptides in the influenza virus receptor binding inhibition assay [17].

Compound^a^	Core, Х (*n*, number of glycine residues in antenna)	Virus	Relative activity

Neu5AcαOBn		(A/Н3N2/29/90)	1(150)^b^
Neu5Acα-sp1-Gly*_n_*-Х-Gly*_n_*-sp1-Neu5Acα	С_2_ (2–4)		1
	С_2_ (5)		3
	С_2_ (6)		6
Neu5Acα-sp1-Gly*_n_*-Х-Gly*_n_*-sp1-Neu5Acα	C_10_ (1–3)		1
	С_10_ (4)		50
	С_10_ (5)		25
6’SLN		(A/H1N1/NIB23)	1(150)^b^
6’SLN-sp2-Gly*_n_*-Х-Gly*_n_*-sp2-6’SLN	OEG (2, 4)		1
	ОEG (5)		1
	ОEG (6)		40
3’SL		(A/H5N2)	1(150)^b^
3’SL-sp3-Gly*_n_*-Х-Gly*_n_*-sp3-3’SL	ОEG (2, 4)		1
	ОEG (5)		1
	ОEG (6)		50

^a^Abbreviations: sp1 = -OCH_2_(p-C_6_H_4_)NHCOCH_2_NH-CO(CH_2_)_4_CO; Bn – benzyl; sp2 = -O(CH_2_)_3_NHCO(CH_2_)_4_CO; 6’SLN = Neu5Acα2-6Galβ1-4GlcNAcβ; sp3 = -NHCOCH_2_NHCO(CH_2_)_4_CO; 3’SL = Neu5Acα2-3Galβ1-4Glcβ*. *^b^Values of IC_50_, μM, for monomeric Neu5AcαOBn, 6'SLN and 3’SL are given in parentheses.

## Experimental

Reagents and solvents were bought from Merck and Sigma–Aldrich. Activated esters BocGlyONSu and BocGly_2_ONSu were prepared as described earlier [9] from glycine or glycylglycine (Acros). Ethylenediamine and 1,10-diaminodecane were supplied from Sigma-Aldrich, and diamine NH_2_(CH_2_CH_2_O)_3_CH_2_CH_2_NH_2_ (**1**) was synthesized from ditosylate TosO(CH_2_CH_2_O)_3_CH_2_CH_2_OTos (Sigma–Aldrich) according to the described methods [7–8].

Silica gel (Kieselgel 60, Merck, Germany) was used for low-pressure column chromatography. Sephadex LH-20 (Pharmacia Biotech, Austria) was employed for gel chromatography. Thin-layer chromatography (TLC) was performed on foil plates covered with silica gel (Kieselgel 60, Merck, Germany).

^1^Н NMR spectra were recorded on a Bruker spectrometer (600, 700, 800 MHz) at 303 K. Chemical shifts (δ) for characteristic signals in ^1^Н NMR spectra are given in ppm and spin–spin coupling constants (*J*) in Hz. The scale of the chemical shifts was calibrated against the signals of residual protons of solvents (CDCl_3_: δ 7.26 ppm; DMSO-*d*_6_: δ 2.50 ppm; D_2_O: δ 4.75 ppm). Mass-spectra were recorded on the time-of-flight spectrometer Vision-2000 (Thermo Bioanalysis, UK) with MALDI with 2,6-dihydroxybenzoic acid as reference. Raman spectra were recorded on a spectrometer Ramanor HG-2S (Jobin Yvon) with the monochromator Anaspec 300S and argon (λ = 514.5 nm, Spectra Physics, model 164-03).

### Synthesis of biantennary oligoglycines

**Protocol 1:** Elongation of the oligoglycine chain (Boc-Gly*_n_*NH-X-NHGly*_n_*-Boc; *n* = 1–7, X = С_2_, С_10_ and OEG). Et_3_N (8 mmol) followed by BocGlyONSu or BocGly_2_ONSu (3 mmol) were added to a solution of diamine (1 mmol) in dimethyl sulfoxide (DMSO; 5 mL). The reaction mixture was stirred until the disappearance of the starting diamine (1–24 h, TLC control) and the solvent was removed under vacuum. The dry residue was suspended in methanol, filtered, dissolved in water, sedimentated with methanol, and dried in vacuo.

**Protocol 2:** Preparation of oligoglycines (HCl·Gly*_n_*NH-X-NHGly*_n_*·HCl; *n* = 1–7, X = С_2_, С_10_ and OEG). The Boc-derivative (0.5 mmol) was dissolved in trifluoroacetic acid (5 mL), the reaction mixture was kept for 2 h at room temperature, co-evaporated with toluene (2 × 10 mL) and 1 M HCl aqueous solution (1–2 mL), and finally with a mixture iPrOH/methanol 1:1 (2 × 10 mL). The obtained product was sedimentated from the aqueous solution by the addition of methanol and dried in vacuo.

### Synthesis of associating glycopeptides

**Protocol 3:** Neu5Acα-sp1-ONp, 3'SL-sp3-ONp or 6'SLN-sp2-ONp (4 μmol) were added to a solution of diamine (1 μmol) in DMSO or saturated aqueous solution of LiBr (200 μL). NEt_3_ (4 μmol) was added until a рН of 8 was reached, and the mixture was stirred for 24 h at room temperature. Exclusion chromatography on Sephadex LH-20 (eluent: 0.1 М solution of NH_3_ in the mixture acetonitrile/water, 1:1). Fractions containing pure product were combined and evaporated. Dry residue was dissolved in water and freeze-dryed.

### Dynamic light scattering experiments

The light scattering of aqueous solutions of biantennary oligoglycines was studied with an analyzer of submicron particle size “Malvern HPPS” (UK). After the preparation of aqueous (Milli-Q) solutions of oigoglycine salts in a concentration of 0.01–0.1 mg/mL, the instrument reading was recorded (*t* = 0, pH < 5). Then, 1–2 equiv of base (0.1 М aqueous solution of NaHCO_3_ or Na_2_CO_3_) per amino group was added to the solution of the analyzed oligoglycine salt (*t* > 0, pH 6–8), and instrument readings were recorded in fixed periods of time. In the case of the formation of large associates (intense opalescence, sedimentation), whose dimensions exceeded the working limit of the instrument, the experiment was stopped.

For experiments with biantennary sialooligoglycines their aqueous (Milli-Q) solutions with a concentration of 0.1 mg/mL were used.

### Scanning force microscopy (SFM)

The samples were imaged with a Nanoscope IIIa instrument (Digital Instruments, USA). Commercial silicon nitride cantilevers with force constants of 0.06, 0.12, and 0.32 Nm^−1^ were used for the measurements in contact mode in liquid cell. Cantilevers with a resonance frequency of about 300 kHz and a force constant of 42 Nm^−1^ were used for the SFM tapping mode in air. Software WSxM (Nanotec Electronica, Spain) was employed for the image treatment. Pure water (Fluka) was used for the preparation of solutions.

**Scanning in air**. 1–2 equiv of 0.1 M of aqueous solution of NaHCO_3_ or Na_2_CO_3_ per amino group (pH ~ 6–8) was added for the deprotonation to a freshly prepared solution of oligoglycine salt (0.1–1.0 mg/mL; pH < 5), and incubated for a specified time period in the range of 0 to 90 min. Then the solution was applied on the freshly cleaved mica or graphite, and kept for a specified period of time within the range of 0 to 10 min. Liquid was removed from the surface by spin coating or in nitrogen flow. Structures formed on the surface were visualized in tapping mode SFM.

**Scanning in liquid cell*.*** A plate of freshly cleaved mica (1 × 1 cm^2^) was placed in a liquid cell. The cell was filled with water (25 μL) and the instrument was set up. Then, water was changed with a freshly prepared solution of deprotonated peptide (see scanning in air above) and the surface was scanned in contact mode SFM in fixed time periods.

**The influenza virus receptor-binding inhibition assay** was carried out as described in [17].

## Supporting Information

File 1 Descriptions of the synthesis of individual compounds.

## References

[R1] 1Tsygankova, S. V.; Chinarev, A. A.; Tuzikov, A. B.; Gambaryan, A. S.; Bovin, N. V. “Self-assembling glycopeptides” presented at the 17th European Carbohydrate Symposium “EuroCarb17”, Tel Aviv, Israel, July 7–11, 2013, P-55.

[2] Tuzikov A B, Chinarev A A, Gambaryan A S, Oleinikov V A, Klinov D V, Matsko N B, Kadykov V A, Ermishov M A, Demin I V, Demin V V (2003). ChemBioChem.

[3] Bovin N V, Tuzikov A B, Chinarev A A (2008). Nanotechnologies in Russia.

[4] Bamford C H, Brown L, Cant E M, Elliott A, Hanby W E, Malcolm B R (1955). Nature.

[5] Crick F H C, Rich A (1955). Nature.

[6] Bovin N V, Tuzikov A B, Chinarev A A, Gambaryan A S (2004). Glycoconjugate J.

[7] Katritzky A R, Singh S K, Meher N K, Doskocz J, Suzuki K, Jiang R, Sommen G L, Ciaramitaro D A, Steel P J (2006). ARKIVOC.

[8] Katritzky A R, Meher N K, Hanci S, Gyanda R, Tala S R, Mathai S, Duran R S, Bernard S, Sabri F, Singh S K (2008). J Polym Sci, Part A: Polym Chem.

[9] Gershkovich A A, Kibirev V C (1992). Chemical synthesis of peptides.

[10] Chinarev A A, Tuzikov A B, Gambaryan A S, Matrosovich M N, Imberty A, Bovin N V, Ynoue Y, Lee Y C, Troy F A (1999). Sialobiology and Other Novel Forms of Glycosylation.

[11] Bovin N V, Chinarev A A, Tuzikov A B (2008). Multiligand constructs. WO Patent.

[12] Krimm S, Bandekar J, Anfinsen C B, Edsall J T, Richards F M Advanced Protein Chemistry.

[13] Gus’kova O A, Khalatur P G, Khokhlov A R, Chinarev A A, Tsygankova S V, Bovin N V (2010). Russ J Bioorg Chem.

[14] Bella J, Puiggali J, Subirana J A (1994). Polymer.

[15] Mammen M, Choi S-K, Whitesides G M (1998). Angew Chem, Int Ed.

[16] Chugunov P A, Chinarev A A, Tuzikov A B, Formanovsky A A, Prokhorov V V, Gambaryan A S, Bovin N V (2009). Mendeleev Commun.

[17] Gambaryan A S, Matrosovich M N (1992). J Virol Methods.

[18] Gambaryan A S, Tuzikov A B, Piskarev V E, Yamnikova S S, Lvov D K, Robertson J S, Bovin N V, Matrosovich M N (1997). Virology.

