# Supplementary material for: Biantennary oligoglycines and glyco-oligoglycines self-associating in aqueous medium
Source: Beilstein J Org Chem. 2014 Jun 17;10:1372–82. doi: 10.3762/bjoc.10.140 (PMC4077438; doi:10.3762/bjoc.10.140)
Supplement: File 1 — Descriptions of the synthesis of individual compounds. [file Beilstein_J_Org_Chem-10-1372-s001.pdf]

Biantennary oligoglycines and glyco-oligoglycines self-associating in aqueous medium

Svetlana V. Tsygankova[1], Alexander A. Chinarev[1], Alexander B. Tuzikov[1], Nikolai Severin[2], Alexey A. Kalachev[3], Juergen P. Rabe[2], Alexandra S. Gambaryan[4], Nicolai V. Bovin[1*]

Address: [1]Shemyakin-Ovchinnikov Institute of Bioorganic Chemistry, ul. Miklukho-Maklaya 16/10 Moscow V-437, 117997, Russia, [2]Department of Physics, Humboldt University Berlin, Newtonstr. 15, D-12489, Berlin, Germany, [3]Plasmachem GmbH, Rudower Chaussee 29, D-12489 Berlin, Germany and [4]M. P. Chumakov Institute of Poliomyelitis and Viral Encephalitides, 142782 Moscow Region, Russia

Email: Nicolai V. Bovin - bovin@carb.ibch.ru

*Corresponding author

Descriptions of the synthesis of individual compounds

Experimental

All reagents and solvents were commercially purchased from Merck and Sigma–Aldrich and further purified according to the standart methods, if necessary. Activated esters BocGlyONSu and BocGly$_2$ONSu were prepared as described earlier [1] from glycine or glycylglycine (Acros). Ethylene diamine and 1,10-diaminodecane were from Sigma–Aldrich, and diamine $NH_2(CH_2CH_2O)_3CH_2CH_2NH_2$ (**1**) was synthesized

from ditosylate $TosO(CH_2CH_2O)_3CH_2CH_2OTos$ (Sigma–Aldrich) according to the described methods [2,3].

Silica gel Kieselgel 60 (Merck, Germany) was used for low-pressure column chromatography; Sephadex LH-20 (Pharmacia Biotech, Austria) was used for gel chromatography. Thin layer chromatography (TLC) was performed on foil plates covered with silica gel (Kieselgel 60, Merck, Germany).

^{1}H NMR spectra were recorded on a Bruker spectrometer (600, 700, 800 MHz) at 303 K. Chemical shifts (δ) for characteristic signals in ^{1}H NMR spectra are given in ppm and spin-spin coupling constants (J) in Hz (the scale of chemical shifts was calibrated by the signals of residual protons of solvents: $CDCl_3$: δ 7.26 ppm; DMSO-d_6: δ 2.50 ppm; D_2O: δ 4.75 ppm). Mass-spectra were recorded on time-of-flight spectrometer Vision-2000 (Thermo Bioanalysis, UK) with MALDI, using 2,6-dihydroxybenzoic acid as reference. Raman spectra were recorded on spectrometer Ramanor HG-2S (Jobin Yvon) with monochromator Anaspec 300S and argon (λ = 514.5 nm, Spectra Physics, model 164-03).

Synthesis of biantennary oligoglycines.

Protocol 1: Elongation of oligoglycine chain (Boc-Gly$_n$NH-X-NHGly$_n$-Boc; n = 1–7, X=C_2, C_{10} and OEG). Triethylamine (Et_3N) (8 mmol) followed by BocGlyONSu or BocGly$_2$ONSu (3 mmol) were added to a solution of diamine (1 mmol) in dimethylsulfoxide (DMSO; 5 mL). Reaction mixture was stirred till the disappearing of the starting diamine (1–24 h, TLC control) and the solvent was removed under a vacuum. Dry residue was suspended in methanol, filtered, dissolved in water, sedimented with methanol and dried in vacuum.

Protocol 2: Preparation of oligoglycines ($HCl*Gly_nNH-X-NHGly_n*HCl$; n = 1–7, X=C_2, C_{10} and OEG). Boc-derivative (0.5 mmol) were dissolved in trifluoroacetic acid (5 mL), reaction mixture was kept for 2 h at room temperature, co-evaporated with toluene (2 × 10 mL), and 1 M HCl aqueous solution (1–2 mL), and finally with a mixture iPrOH-methanol, 1:1 (2 × 10 mL). The obtained product was sedimented from aqueous solution by addition of methanol and dried in vacuum.

Synthesis of oligoglycine derivatives with OEG core.

$BocGly_2-NH(CH_2CH_2O)_3CH_2CH_2NH-Gly_2Boc$ (**2**) was prepared according to Protocol 1 from diamine **1** (300 mg, 1.56 mmol) and $BocGly_2ONSu$ (1.5 g, 4.68 mmol) in 10 mL dioxane. The product was purified by silica gel column chromatography (eluting solvent: iPrOH–EtOAc–H_2O, 2:7:1) to afford 635 mg of **2** (66%) as colorless oil. TLC (eluent: iPrOH–EtOAc–H_2O, 2:3:1): R_f=0.52. ^{1}H-NMR (D_2O): 3.90 (s, 4H, 2×CH_2 Gly); 3.78 (s, 4H, 2×CH_2 Gly); 3.66–3.63 (m, 8H, 4×CH_2O core); 3.61–3.57 (m, 4H, 2×CH_2O core); 3.41–3.37 (m, 4H, 2×CH_2NH core); 1.41 (s, 18H, 2×C(CH_3)$_3$). MS: *m/z*: 621 $(M)^+$, 644 $(M+Na)^+$. Found, %: C 50.25; H 7.82; N 13.50, $C_{26}H_{48}N_6O_{11}$. Calculated, %: C 50.31; H 7.79; N 13.54.

$HCl*Gly_2-NH(CH_2CH_2O)_3CH_2CH_2NH-Gly_2*HCl$ (**3**) was prepared according to Protocol 2 from *Boc*-derivative **2** (300 mg, 0.48 mmol) as white powder (274 mg, 88%). TLC (eluent: ethanol–25% aqueous NH_3, 3:1): R_f=0.6. ^{1}H-NMR (D_2O): 3.95 (s, 4H, 2×CH_2 Gly); 3.84 (s, 4H, 2×CH_2 Gly); 3.66–3.64 (m, 8H, 4×CH_2O core); 3.60 (t, 4H, J = 5.4 Hz, 2×CH_2O core); 3.40 (t, 4H, J = 5.4 Hz, 2×CH_2NH core).

BocGly$_4$NH(CH$_2$CH$_2$O)$_3$CH$_2$CH$_2$NHGly$_4$Boc (**4**) was prepared according to Protocol 1 from diamine **3** (205 mg, 0.42 mmol) and BocGly$_2$ONSu (411 mg, 1.25 mmol) in 10 mL dioxane. It was obtained 270 mg of **4** as white powder (80%). TLC (eluent: iPrOH–EtOAc–H_2O, 2:3:1): R_f=0.23. ^{1}H-NMR (D_2O): 4.02 (s, 4H, 2×C*H₂* Gly); 4.00 (s, 4H, 2×C*H₂* Gly); 3.94 (s, 4H, 2×C*H₂* Gly); 3.85 (s, 4H, 2×C*H₂* Gly); 3.71–3.68 (m, 8H, 4×C*H₂*O core); 3.64 (t, 4H, *J* = 5.4 Hz, 2×C*H₂*O core); 3.43 (t, 4H, *J* = 5.4 Hz, 2×C*H₂*NH core); 1.45 (s, 18H, 2×C(C*H₃*)$_3$). MS: *m/z*: 849 (M)$^+$, 872 (M+Na)$^+$. Found, %: C 48.05; H 7.21; N 16.45, $C_{34}H_{60}N_{10}O_{15}$. Calculated, %: C 48.11; H 7.12; N 16.50.

*HCl*Gly$_4$-NH(CH$_2$CH$_2$O)$_3$CH$_2$CH$_2$NH-Gly$_4$*HCl* (**5**) was prepared according to Protocol 2 from *Boc*-derivative **4** (80 mg, 1.2 μmol) as white powder (65 mg, 95%). TLC (eluent: ethanol–25% aqueous NH_3, 3:1): R_f=0.4. ^{1}H-NMR (D_2O): 4.08 (s, 4H, 2×C*H₂* Gly); 4.02 (s, 4H, 2×C*H₂* Gly); 3.94 (s, 4H, 2×C*H₂* Gly); 3.92 (s, 4H, 2×C*H₂* Gly); 3.71–3.69 (m, 8H, 4×C*H₂*O core); 3.64 (t, 4H, *J* = 5.4 Hz, 2×C*H₂*O core); 3.43 (t, 4H, *J* = 5.4 Hz, 2×C*H₂*NH core).

BocGly$_5$-NH(CH$_2$CH$_2$O)$_3$CH$_2$CH$_2$NH-Gly$_5$Boc (**6**) was prepared according to Protocol 1 from diamine **5** (10 mg, 13.8 mmol) and BocGlyONSu (10.6 mg, 41.6 μmol) in DMSO. The mixture was stirred for 1 h at 60 °C, evaporated, and DMSO was removed in vacuum. The obtained white residue was suspended in MeOH and the precipitate was washed with MeOH and dried in vacuum. It was obtained 11.2 mg of **6** as white powder (84%). TLC (eluent: iPrOH–EtOAc–H_2O, 2:3:1): R_f=0.18. ^{1}H-NMR (DMSO-d_6): 8.12–8.08 (m, 4H, 4×N*H* Gly); 8.05–8.00 (m, 2H, 2×N*H* Gly); 8.00–7.95 (m, 2H, 2×N*H* Gly); 7.80–7.75 (m, 2H, 2×N*H* Gly); 6.99–6.95 (m, 2H, 2×*NH* core); 3.77 (d, 4H, J_{NH} = 5.2 Hz, 2×C*H₂* Gly); 3.75 (d, 4H, J_{NH} = 5.2 Hz, 2×C*H₂* Gly); 3.73 (d,

4H, J_{NH} = 5.3 Hz, 2×CH_2 Gly); 3.68 (d, 4H, J_{NH} = 5.2 Hz, 2×CH_2 Gly); 3.59 (d, 4H, J_{NH} = 5.2 Hz, 2×CH_2 Gly); 3.52–3.49 (m, 8H, 4×CH_2O core); 3.43–3.40 (m, 4H, 2×CH_2O core); 3.23–3.20 (m, 4H, 2×CH_2NH core); 1.35 (s, 18H, 2×C(CH_3)$_3$). MS: *m/z*: 963 $(M)^+$, 986 $(M+Na)^+$. Found, %: C 47.33; H 6.94; N 17.41. $C_{38}H_{66}N_{12}O_{17}$. Calculated, %: C 47.40; H 6.91; N 17.45.

*HCl*Gly$_5$-NH(CH$_2$CH$_2$O)$_3$CH$_2$CH$_2$NH-Gly$_5$*HCl* (**7**) was prepared according to Protocol 2 from *Boc*-derivative **6** (10 mg, 10 μmol) as white powder (8 mg, 92%). TLC (eluent: ethanol–H_2O–AcOH–pyridine, 3:1:1:1): R_f=0.46. ^{1}H-NMR (D_2O): 4.05 (s, 4H, 2×CH_2 Gly); 3.99 (s, 4H, 2×CH_2 Gly); 3.97 (s, 4H, 2×CH_2 Gly); 3.91 (s, 4H, 2×CH_2 Gly); 3.89 (s, 4H, 2×CH_2 Gly); 3.66–3.64 (m, 8H, 4×CH_2O core); 3.62 (t, 4H, *J* = 5.4 Hz, 2×CH_2O core); 3.40 (t, 4H, *J* = 5.4 Hz, 2×CH_2NH core).

BocGly$_6$-NH(CH$_2$CH$_2$O)$_3$CH$_2$CH$_2$NH-Gly$_6$Boc (**8**) was prepared according to Protocol 1 from diamine **5** (5 mg, 6.9 μmol) and BocGly$_2$ONSu (6.8 mg, 20.8 μmol) in DMSO. The mixture was stirred for 1 h at 80°C, evaporated and DMSO was removed in vacuum. The obtained white residue was suspended in MeOH and the precipitate was washed with MeOH and dried in vacuum. It was obtained 6 mg (81%) of **8** as white powder.[♦] MS: *m/z*: 1077 $(M)^+$, 1100 $(M+Na)^+$. Found, %: C 46.80; H 6.79; N 18.18. $C_{42}H_{72}N_{14}O_{19}$. Calculated, %: C 46.83; H 6.74; N 18.21.

*HCl*Gly$_6$-NH(CH$_2$CH$_2$O)$_3$CH$_2$CH$_2$NH-Gly$_6$*HCl* (**9**) was prepared according to Protocol 2 from *Boc*-derivative **8** (5 mg, 4.6 μmol) as white powder (4 mg, 91%). TLC (eluent: ethanol–H_2O–AcOH–pyridine, 3:1:1:1): R_f=0.36. ^{1}H-NMR (D_2O): 4.10 (s, 4H,

---

♦ Due to very low solubility of the substance we failed to get a well resolved ^{1}H-NMR spectrum. Spectrum was recorded for the corresponding hydrochloride.

2×CH_2 Gly); 4.05 (s, 4H, 2×CH_2 Gly); 4.04 (s, 4H, 2×CH_2 Gly); 4.02 (s, 4H, 2×CH_2 Gly); 3.96 (s, 4H, 2×CH_2 Gly); 3.94 (s, 4H, 2×CH_2 Gly); 3.73–3.71 (m, 8H, 4×CH_2O core); 3.66 (t, 4H, J = 5.4 Hz, 2×CH_2O core); 3.45 (t, 4H, J = 5.4 Hz, 2×CH_2NH core).

BocGly$_7$-NH(CH$_2$CH$_2$O)$_3$CH$_2$CH$_2$NH-Gly$_7$Boc (**10**) A solution of saturated aqueous LiCl (0.5 mL) followed by BocGlyONSu (7 mg, 27 μmol) was added to a solution of diamine **9** (4.3 mg, 4.5 μmol) in water (0.1 mL). After addition of Li_2CO_3 (1.35 mg, 18 μmol) the mixture was stirred for 24 h at room temperature, then diluted with water (3 mL). The white precipitate was filtered off, washed with water and MeOH (4 × 1 mL) and dried in vacuum to afford 4 mg (75%) of *Boc*-derivative **10** as white powder♦. MS: *m/z*: 1192 $(M)^+$, 1215 $(M+Na)^+$. Found, %: C 46,35; H 6.70; N 18.78. $C_{46}H_{78}N_{16}O_{21}$. Calculated, %: C 46.38; H 6.60; N 18.81.

*HCl*Gly$_7$-NH(CH$_2$CH$_2$O)$_3$CH$_2$CH$_2$NH-Gly$_7$*HCl* (**11**) was prepared according to Protocol 2 from *Boc*-derivative **10** (4 mg, 3.3 μmol) as white powder (3.1 g, 88%). ^{1}H NMR (D_2O): 4.10 (s, 4H, 2×CH_2 Gly); 4.05–4.01 (m, 16H, 8×CH_2 Gly); 3.96 (s, 4H, 2×CH_2 Gly); 3.94 (s, 4H, 2×CH_2 Gly); 3.73–3.71 (m, 8H, 4×CH_2O core); 3.65 (t, 4H, J = 5.4 Hz, 2×CH_2O core); 3.44 (t, 4H, J = 5.4 Hz, 2×CH_2NH core).

Synthesis of oligoglycine derivatives with core C_2.

BocGly$_2$-NH(CH$_2$)$_2$NH-Gly$_2$Boc (**12**) was prepared according to Protocol 1 from ethylenediamine (0.1 g, 1.67 mmol) and BocGly$_2$ONSu (1.6 g, 5 mmol) in DMSO. After re-crystallization from 20 mL of the mixture methanol–water (1:1) it was obtained 0.73 g (88%) of *Boc*-derivative **12**. Melting range 215–217 °C. TLC (eluent: iPrOH–EtOAc–H_2O, 2:3:1): R_f=0.65. ^{1}H-NMR (DMSO-d_6): 8.00 (br. s, 2H, 2×NH Gly);

7.82 (br. s, 2H, 2×N*H* Gly); 6.93 (br. s, 2H, 2×CH_2N*H* core); 3.67 (d, 4H, J_{NH} = 5.2 Hz, 2×CH_2 Gly); 3.58 (d, 4H, J_{NH} = 5.2 Hz, 2×CH_2 Gly); 3.10 (br. s, 4H, 2×CH_2NH core); 1.38 (s, 18H, 2×C(CH_3)$_3$). MS: *m/z*: 489 $(M)^+$, 512 $(M+Na)^+$. Found, %: C 49.05; H 7.59; N 17.09. $C_{20}H_{36}N_6O_8$. Calculated, %: C 49.18; H 7.43; N 17.21.

*HCl*Gly$_2$-NH(CH$_2$)$_2$NH-Gly$_2$*HCl* (**13**) was prepared according to Protocol 2 from *Boc*-derivative **12** (1.42 g, 2.9 mmol) as white powder (1.04 g, 99%). TLC (eluent: iPrOH–acetonitrile–H_2O, 4:3:2): R_f=0.30. ^{1}H-NMR (D_2O): 3.98 (s, 4H, 2×CH_2 Gly); 3.92 (s, 4H, 2×CH_2 Gly); 3.37 (s, 4H, 2×CH_2NH core).

BocGly$_3$-NH(CH$_2$)$_2$NH-Gly$_3$Boc (**14**) was prepared according to Protocol 1 from diamine **13** (30 mg, 0.08 mmol) and BocGlyONSu (64 mg, 0.25 mmol) in DMSO. After purification it was obtained 40 mg (83%) of *Boc*-derivative **14**. TLC (eluent: iPrOH–EtOAc–H_2O, 2:3:1): R_f=0.50. ^{1}H-NMR (DMSO-d_6): 8.10–8.02 (m, 4H, 4×N*H* Gly); 7.79 (br. s, 2H, 2×N*H* Gly); 6.93 (br. s, 2H, 2×CH_2N*H* core); 3.75 (d, 4H, J_{NH} = 5.6 Hz, 2×CH_2 Gly); 3.67 (d, 4H, J_{NH} = 5.6 Hz, 2×CH_2 Gly); 3.58 (d, 4H, J_{NH} = 5.5 Hz, 2×CH_2 Gly); 3.11 (br. s, 4H, 2×CH_2NH core); 1.38 (s, 18H, 2×C(CH_3)$_3$). MS: *m/z*: 603 $(M)^+$, 626 $(M+Na)^+$. Found, %: C 47.79; H 7.09; N 18.53. $C_{24}H_{42}N_8O_{10}$. Calculated, %: C 47.83; H 7.02; N 18.59.

*HCl*Gly$_3$-NH(CH$_2$)$_2$NH-Gly$_3$*HCl* (**15**) was prepared according to Protocol 2 from *Boc*-derivative **14** (30 mg, 0.05 mmol) as white powder (21.6 mg, 91%). TLC (eluent: iPrOH–acetonitrile–water, 4:3:2): R_f=0.10. ^{1}H-NMR (D_2O): 4.01 (s, 4H, 2×CH_2 Gly); 3.98 (s, 4H, 2×CH_2 Gly); 3.91 (s, 4H, 2×CH_2 Gly); 3.37 (s, 4H, 2×CH_2NH core).

BocGly$_4$-NH(CH$_2$)$_2$NH-Gly$_4$Boc (**16**) was prepared according to Protocol 1 from diamine **13** (400 mg, 1.1 mmol) and BocGly$_2$ONSu (1.08 g, 3.3 mmol) in DMSO (7 mL). The mixture was stirred for 1 h at 60 °C, evaporated, and DMSO was removed in vacuum. The obtained white residue was suspended in MeOH and the precipitate was washed with MeOH and dried in vacuum. It was obtained 0.72 g of **16** as white powder (92%). MS: *m/z*: 717 $(M)^+$, 740 $(M+Na)^+$. Found, %: C 46.89; H 6.84; N 19.49. $C_{28}H_{48}N_{10}O_{12}$. Calculated, %: C 46.92; H 6.75; N 19.54.

*HCl*Gly$_4$-NH(CH$_2$)$_2$NH-Gly$_4$*HCl* (**17**) was prepared according to Protocol 2 from *Boc*-derivative **16** (0.7 g, 0.98 mmol) as white powder (0.52 g, 91%). TLC (eluent: ethanol-25% aqueous NH_3, 3:1): R_f=0.40. ^{1}H-NMR (D_2O): 4.09 (s, 4H, 2×CH_2 Gly); 4.03 (s, 4H, 2×CH_2 Gly); 3.92 (s, 4H, 2×CH_2 Gly); 3.90 (s, 4H, 2×CH_2 Gly); 3.37 (s, 4H, 2×CH_2NH core).

BocGly$_5$-NH(CH$_2$)$_2$NH-Gly$_5$Boc (**18**) was prepared according to Protocol 1 from diamine **17** (180 mg, 0.3 mmol) and BocGlyONSu (235 mg, 0.9 mmol) in DMSO (3 mL). The mixture was stirred for 1 h at 60 °C, evaporated, and DMSO was removed in vacuum. The obtained white residue was suspended in MeOH and the precipitate was washed with MeOH and dried in vacuum. It was obtained 155 mg of *Boc*-derivative **18** as white powder (62%)♦. MS: *m/z*: 831 $(M)^+$, 854 $(M+Na)^+$. Found, %: C 46.20, H 6.77, N 20.18. $C_{32}H_{54}N_{12}O_{14}$. Calculated, %: C 46.26, H 6.55, N 20.23.

*HCl*Gly$_5$-NH(CH$_2$)$_2$NH-Gly$_5$*HCl* (**19**) was prepared according to Protocol 2 from *Boc*-derivative **18** (155 mg, 0.18 mmol) as white powder (60 mg, 46%). TLC (eluent: ethanol–25% aqueous NH_3, 3:1): R_f=0.33. ^{1}H-NMR (D_2O): 4.14 (s, 4H, 2×CH_2 Gly);

4.09 (s, 4H, 2×CH_2 Gly); 4.06 (s, 4H, 2×CH_2 Gly); 3.98 (s, 4H, 2×CH_2 Gly); 3.96 (s, 4H, 2×CH_2 Gly); 3.40 (s, 4H, 2×CH_2NH core).

BocGly$_6$-NH(CH$_2$)$_2$NH-Gly$_6$Boc (**20**) was prepared according to Protocol 1 from diamine **17** (14 mg, 0.024 mmol) and BocGly$_2$ONSu (23.5 mg, 0.071 mmol) in DMSO (0.4 mL). The mixture was stirred for 1 h at 80 °C, evaporated, and DMSO was removed in vacuum. The obtained white residue was suspended in MeOH and the precipitate was washed with MeOH and dried in vacuum. It was obtained 19 mg of *Boc*-derivative **20** as white powder (76%)♦. MS: *m/z*: 945 $(M)^+$, 968 $(M+Na)^+$. Found, %: C 45.69; H 6.43; N 20.71. $C_{36}H_{60}N_{14}O_{16}$. Calculated, %: C 45.76; H 6.40; N 20.75.

*HCl*Gly$_6$-NH(CH$_2$)$_2$NH-Gly$_6$*HCl* (**21**) was prepared according to Protocol 2 from *Boc*-derivative **20** (19 mg, 0.018 mmol) as white powder (10 mg, 68%). TLC (eluent: ethanol-25% aqueous NH_3, 3:1): R_f=0.23. ^{1}H-NMR (D_2O): 4.09 (s, 4H, 2×CH_2 Gly); 4.05 (s, 4H, 2×CH_2 Gly); 4.04 (s, 4H, 2×CH_2 Gly); 4.01 (s, 4H, 2×CH_2 Gly); 3.94 (s, 4H, 2×CH_2 Gly); 3.92 (s, 4H, 2×CH_2 Gly); 3.37 (s, 4H, 2×CH_2NH core).

Synthesis of oligoglycine derivatives with C_{10} core.

BocGly-NH(CH$_2$)$_{10}$NH-GlyBoc (**22**) was prepared according to Protocol 1 from 1,10-diaminodekane (1.2 g, 6.9 mmol) and BocGlyONSu (5.35 g, 20.9 mmol) in DMSO (20 mL). The mixture was stirred for 1 h at 60 °C, evaporated, and DMSO was removed in vacuum. The obtained white residue was suspended in MeOH and the precipitate was washed with MeOH and dried in vacuum. It was obtained 3.22 g of *Boc*-derivative **22** as white powder (95%). TLC (eluent: EtOAc): R_f=0.3. ^{1}H-NMR (DMSO-*d*$_6$): 7.65 (br. s, 2H, 2×N*H* Gly); 6.83 (br. s, 2H, 2×CH$_2$N*H* core); 3.48 (d, 4H, J_{NH} = 5.8

Hz, 2×CH_2 Gly); 3.05–3.01 (m, 4H, 2×CH_2NH core); 1.39–1.35 (m, 22H, 2×CH_2 core, 2×C(CH_3)$_3$); 1.25–1.22 (m, 12H, 6×CH_2 core). MS: *m/z*: 487 $(M)^+$, 510 $(M+Na)^+$. Found, %: C 59.19; H 9.69; N 11.48. $C_{24}H_{46}N_4O_6$. Calculated, %: C 59.23; H 9.53; N 11.51.

*HCl*Gly-NH(CH$_2$)$_{10}$NH-Gly*HCl* (**23**) was prepared according to Protocol 2 from *Boc*-derivative **22** (2.89 g, 5.9 mmol) as white powder (1.74 g, 82%). TLC (eluent: ethanol–25% aqueous NH_3, 1:1): R_f=0.83. ^{1}H-NMR (D_2O): 3.80 (s, 4H, 2×CH_2 Gly); 3.30 (t, 4H, J = 7.0 Hz, 2×CH_2NH core); 1.55–1.50 (m, 4H, 2×CH_2CH$_2$NH core); 1.35–1.30 (m, 12H, 6×CH_2 core).

BocGly$_2$-NH(CH$_2$)$_{10}$NH-Gly$_2$Boc (**24**) was prepared according to Protocol 1 from 1,10-diaminodekane (0.95 g, 5.5 mmol) and BocGly$_2$ONSu (5.45 g, 16.5 mmol) in DMSO (20 mL). The mixture was stirred for 1 h at 60 °C, evaporated, and DMSO was removed in vacuum. The obtained white residue was suspended in MeOH and the precipitate was washed with MeOH and dried in vacuum. It was obtained 3.0 g of *Boc*-derivative **24** as white powder (91%). TLC (eluent: iPrOH–EtOAc–H_2O, 2:3:1): R_f=0.70. ^{1}H-NMR (DMSO-d_6): 7.97 (br.s, 2H, 2×NH Gly); 7.62 (br.s, 2H, 2×NH Gly); 7.00 (t, 2H, J = 5.5 Hz, 2×CH$_2$NH core); 3.65 (d, 4H, J_{NH} = 5.5 Hz, 2×CH_2 Gly); 3.55 (d, 4H, J_{NH} = 5.5 Hz, 2×CH_2 Gly); 3.06–3.02 (m, 4H, 2×CH_2NH core); 1.47–1.37 (m, 22H, 2×CH_2 core, 2×C(CH_3)$_3$); 1.32–1.25 (m, 12H, 6×CH_2 core). MS: *m/z*: 601 $(M)^+$, 624 $(M+Na)^+$. Found, %: C 55.94; H 8.81; N 13.93. $C_{28}H_{52}N_6O_8$. Calculated, %: C 55.98; H 8.72; N 13.99.

*HCl*Gly$_2$-NH(CH$_2$)$_{10}$NH-Gly$_2$*HCl* (**25**) was prepared according to Protocol 2 from *Boc*-derivative **24** (2.9 g, 4.8 mmol) as white powder (2.22 g, 98%). TLC (eluent: ethanol–25% aqueous NH_3, 1:1): R_f=0.62. ^{1}H-NMR (D_2O): 3.96 (s, 4H, 2×CH_2 Gly); 3.93 (s, 4H, 2×CH_2 Gly); 3.22 (t, 4H, J = 7.0 Hz, 2×CH_2NH core); 1.52–1.48 (m, 4H, 2×CH_2CH$_2$NH core); 1.35–1.27 (m, 12H, 6×CH_2 core).

BocGly$_3$-NH(CH$_2$)$_{10}$NH-Gly$_3$Boc (**26**) was prepared according to Protocol 1 from diamine **23** (0.9 g, 2.5 mmol) and BocGly$_2$ONSu (2.5 g, 7.5 mmol) in DMSO (20 mL). The mixture was stirred for 1 h at 60 °C, evaporated, and DMSO was removed in vacuum. The obtained white residue was suspended in MeOH and the precipitate was re-crystallized from 20 mL of the mixture methanol–water (1:1), was washed with MeOH and dried in vacuum. It was obtained 1.77 g of *Boc*-derivative **26** as colorless crystals (99%). Melting range 217–221 °C. TLC (eluent: iPrOH–EtOAc–H_2O, 2:3:1): R_f=0.54. ^{1}H-NMR (DMSO-d_6): 8.08–8.02 (m, 4H, 4×NH Gly); 7.65 (br. s, 2H, 2×NH Gly); 6.97 (t, 2H, J = 5.4 Hz, 2×CH$_2$NH core); 3.72 (d, 4H, J_{NH} = 5.7 Hz, 2×CH_2 Gly); 3.64 (d, 4H, J_{NH} = 5.7 Hz, 2×CH_2 Gly); 3.60 (d, 4H, J_{NH} = 5.7 Hz, 2×CH_2 Gly); 3.05–3.01 (m, 4H, 2×CH_2NH core); 1.45–1.33 (m, 22H, 2×CH_2 core, 2×C(CH_3)$_3$); 1.25–1.17 (m, 12H, 6×CH_2 core). MS: *m/z*: 715 $(M)^+$, 738 $(M+Na)^+$. Found, %: C 53.71; H 8.25; N 15.54. $C_{32}H_{58}N_8O_{10}$. Calculated, %: C 53.77; H 8.18; N 15.67.

*HCl*Gly$_3$-NH(CH$_2$)$_{10}$NH-Gly$_3$*HCl* (**27**) was prepared according to Protocol 2 from *Boc*-derivative **26** (1.7 g, 2.3 mmol) as white powder (1.12 g, 98%). TLC (eluent: ethanol–25% aqueous NH_3, 1:1): R_f=0.44. ^{1}H-NMR (D_2O): 4.02 (s, 4H, 2×CH_2 Gly); 3.93 (s, 4H, 2×CH_2 Gly); 3.90 (s, 4H, 2×CH_2 Gly); 3.22 (t, 4H, J = 7.0 Hz, 2×CH_2NH core); 1.50 (m, 4H, 2×CH_2CH$_2$NH core); 1.35–1.27 (m, 12H, 6×CH_2 core).

BocGly$_4$-NH(CH$_2$)$_{10}$NH-Gly$_4$Boc (**28**) was prepared according to Protocol 1 from diamine **25** (2.22 g, 4.7 mmol) and BocGly$_2$ONSu (4.6 g, 14 mmol) in DMSO (30 mL). The mixture was stirred for 1 h at 60 °C, evaporated, and DMSO was removed in vacuum. The obtained white residue was suspended in MeOH and the precipitate was washed with MeOH and dried in vacuum. It was obtained 3.75 g of *Boc*-derivative **28** as white powder (96%). TLC (eluent: iPrOH–EtOAc–H_2O, 2:3:1): R_f=0.21. ^{1}H-NMR (DMSO-d_6): 8.15 (t, 2H, J = 5.6 Hz, 2×N*H* Gly); 8.05–8.00 (m, 4H, 4×N*H* Gly); 7.67 (t, 2H, J = 5.7 Hz, 2×N*H* Gly); 6.97 (br. s, 2H, 2×CH_2N*H* core); 3.75 (d, 4H, J_{NH} = 5.6 Hz, 2×C*H$_2$* Gly); 3.72 (d, 4H, J_{NH} = 5.6 Hz, 2×C*H$_2$* Gly); 3.65 (d, 4H, J_{NH} = 5.8 Hz, 2×C*H$_2$* Gly); 3.58 (d, 4H, J_{NH} = 5.6 Hz, 2×C*H$_2$* Gly); 3.06–3.01 (br. s, 4H, 2×C*H$_2$*NH core); 1.47–1.33 (m, 22H, 2×C*H$_2$*CH_2NH core, 2×C(C*H$_3$*)$_3$); 1.25–1.07 (m, 12H, 6×C*H$_2$* core). MS: *m/z*: 829 $(M)^+$, 852 $(M+Na)^+$. Found, %: C 52.09; H 7.87; N 16.84. $C_{36}H_{64}N_{10}O_{12}$. Calculated, %: C 52.16; H 7.78; N 16.90.

*HCl*Gly$_4$-NH(CH$_2$)$_{10}$NH-Gly$_4$*HCl* (**29**) was prepared according to Protocol 2 from *Boc*-derivative **28** (3.75 g, 4.5 mmol) as white powder (2.53 g, 80%). TLC (eluent: ethanol–25% aqueous NH_3, 1:1): R_f=0.10. ^{1}H-NMR (D_2O): 4.13 (s, 4H, 2×C*H$_2$* Gly); 4.03 (s, 4H, 2×C*H$_2$* Gly); 3.99 (s, 4H, 2×C*H$_2$* Gly); 3.94 (s, 4H, 2×C*H$_2$* Gly); 3.21 (t, 4H, J = 7.0 Hz, 2×C*H$_2$*NH core); 1.50 (m, 4H, 2×C*H$_2$*CH_2NH core); 1.35–1.27 (m, 12H, 6×CH_2 core). Found, %: C 44.45; H 7.18; Cl 10.23; N 19.49. $C_{26}H_{50}Cl_2N_{10}O_8$. Calculated, %: C 44.50; H 7.13; Cl 10.13; N 19.97.

BocGly$_5$-NH(CH$_2$)$_{10}$NH-Gly$_5$Boc (**30**) was prepared according to Protocol 1 from diamine **29** (300 mg, 0.42 mmol) and BocGly$_2$ONSu (330 mg, 1.28 mmol) in DMSO

(3 mL). The mixture was stirred for 1 h at 60 °C, evaporated, and DMSO was removed in vacuum. The obtained white residue was suspended in MeOH and the precipitate was washed with MeOH and dried in vacuum. It was obtained 390 mg of *Boc*-derivative **30** as white powder (97%)[♦]. MS: *m/z*: 943 $(M)^+$, 966 $(M+Na)^+$. Found, %: C 50.84; H 7.57; N 17.78. $C_{40}H_{70}N_{12}O_{14}$. Calculated, %: C 50.94; H 7.48; N 17.82.

*HCl*Gly*$_5$*NH-(CH*$_2$*)*$_{10}$*-NHGly*$_5$**HCl* (**31**) was prepared according to Protocol 2 from *Boc*-derivative **30** (390 mg, 0.41 mmol) as white powder (285 mg, 85%) ^{1}H-NMR (D_2O): 4.13 (s, 4H, 2×C*H*$_2$ Gly); 4.08 (s, 4H, 2×C*H*$_2$ Gly); 4.03 (s, 4H, 2×C*H*$_2$ Gly); 3.99 (s, 4H, 2×C*H*$_2$ Gly); 3.94 (s, 4H, 2×C*H*$_2$ Gly); 3.22 (t, 4H, *J* = 7.0 Hz, 2×C*H*$_2$NH core); 1.50 (m, 4H, 2×C*H*$_2$CH$_2$NH core); 1.35–1.27 (m, 12H, 6×C*H*$_2$ core). Found, %: C 44.05; H 6.95; Cl 8.95; N 20.49. $C_{30}H_{56}Cl_2N_{12}O_{10}$. Calculated, %: C 44.17; H 6.87; Cl 8.71; N 20.61.

Synthesis of associating glycopeptides[‡].

Protocol 3: Neu5Acα-sp1-ONp, 3'SL-sp3-ONp or 6'SLN-sp2-ONp (4 μmol) were added to a solution of diamine (1 μmol) in DMSO or saturated aqueous solution of LiBr (200 μl). NEt_3 (4 μmol) was added until pH 8 was reached and the mixture was stirred for 24 h at room temperature. Exclusion chromatography on Sephadex LH-20 (eluent: 0.1 M solution of NH_3 in the mixture acetonitrile–water, 1:1). Fractions containing pure product were combined and evaporated. Dry residue was dissolved in water and freeze dried.

[‡] Descriptions of glycopeptides with the shorts antenna (n<4) are omited here as not having practical interest.

Neu5Acα-sp1-Gly$_5$-NH(CH$_2$)$_2$NH-Gly$_5$-sp1-Neu5Acα (**32**) was prepared according to Protocol 3 from diamine **19**, Neu5Acα-sp1-ONp and NEt_3 in DMSO. Yield: 68%. ^{1}H-NMR (D_2O): 7.42 (m, 8H, 2×*Ar*); 4.75 (d, J_{hem} = 11.1 Hz, 2H, 2×ArC*H$_2$*); 4.55 (d, J_{hem} = 11.1 Hz, 2H, 2×ArC*H$_2$*); 4.06 (s, 4H, 2×C*H$_2$* Gly); 4.02–3.98 (s, 12H, 6×C*H$_2$* Gly); 3.96 (s, 4H, 2×C*H$_2$* Gly); 3.90 (s, 4H, 2×C*H$_2$* Gly); 3.87 (dd, $J_{9b,9a}$ = 12.0 Hz, $J_{8,9a}$ = 2.3 Hz, 2H, 2×H-9a Neu5Acα); 3.84 (dd ≈ t, *J* = 10.0 Hz, 2H, 2×H-5 Neu5Acα); 3.79 (ddd, $J_{9a,8}$ = 2.3 Hz, $J_{9b,8}$ = 6.1 Hz, $J_{7,8}$ = 9.1 Hz, 2H, 2×H-8 Neu5Acα); 3.74 (dd, $J_{7,6}$ = 1.6 Hz, $J_{5,6}$ = 10.5 Hz, 2H, 2×H-6 Neu5Acα); 3.70 (ddd, $J_{5,4}$ = 10.0 Hz, $J_{3eq,4}$ = 4.6 Hz, $J_{3ax,4}$ = 11.8 Hz, 2H, 2×H-4 Neu5Acα); 3.65 (dd, $J_{8,9b}$ = 6.0 Hz, $J_{9a,9b}$ = 12.0 Hz, 2H, 2×H-9b Neu5Acα); 3.61 (dd, $J_{8,7}$ = 9.1 Hz, $J_{6,7}$ = 1.6 Hz, 2H, 2×H-7 Neu5Acα); 3.33 (s, 4H, 2×C*H$_2$* core); 2.79 (dd, $J_{3ax,3eq}$ = 12.4 Hz, $J_{4,3eq}$ = 4.6 Hz, 2H, 2×H-3$_{eq}$ Neu5Acα); 2.37 (m, 8H, 4×COC*H$_2$*CH$_2$ sp); 2.05 (s, 6H, 2×NCOC*H$_3$*); 1.70 (dd ≈ t, *J* = 12.1 Hz, 2H, 2×H-3$_{ax}$ Neu5Acα); 1.67 (m, 8H, 4×COCH$_2$C*H$_2$* sp). MS: *m/z*: 1793 $(M)^+$, 1816 $(M+Na)^+$, 1832 $(M+K)^+$.

Neu5Acα-sp1-Gly$_6$-NH(CH$_2$)$_2$NH-Gly$_6$-sp1-Neu5Acα (**33**) was prepared according to Protocol 3 from diamine **21**, Neu5Acα-sp1-ONp and NEt_3 in saturated aqueous solution of LiBr. Yield: 92%. ^{1}H-NMR (D_2O): 7.45 (m, 8H, 2×*Ar*); 4.75 (d, J_{hem} = 11.1 Hz, 2H, 2×ArC*H$_2$*); 4.55 (d, J_{hem} = 11.1 Hz, 2H, 2×ArC*H$_2$*); 4.07 (s, 4H, 2×C*H$_2$* Gly); 4.00 (s, 16H, 8×C*H$_2$* Gly); 3.96 (s, 4H, 2×C*H$_2$* Gly); 3.90 (s, 4H, 2×C*H$_2$* Gly); 3.87 (dd, $J_{9b,9a}$ = 11.9 Hz, $J_{8,9a}$ = 2.3 Hz, 2H, 2×H-9a Neu5Acα); 3.84 (dd ≈ t, *J* = 10.0 Hz, 2H, 2×H-5 Neu5Acα); 3.79 (ddd, $J_{9a,8}$ = 2.3 Hz, $J_{9b,8}$ = 6.1 Hz, $J_{7,8}$ = 9.1 Hz, 2H, 2×H-8 Neu5Acα); 3.74 (dd, $J_{7,6}$ = 1.4 Hz, $J_{5,6}$ = 10.8 Hz, 2H, 2×H-6 Neu5Acα); 3.70 (ddd, $J_{5,4}$ = 10.0 Hz, $J_{3eq,4}$ = 4.6 Hz, $J_{3ax,4}$ = 11.8 Hz, 2H, 2×H-4 Neu5Acα)*; 3.65* (dd, $J_{8,9b}$ =

6.0 Hz, $J_{9a,9b}$ = 11.9 Hz, 2H, 2×H-9b Neu5Acα); 3.62 (dd, $J_{8,7}$ = 9.1 Hz, $J_{6,7}$ = 1.4 Hz, 2H, 2×H-7 Neu5Acα); 3.33 (s, 4H, 2×CH_2 core); 2.79 (dd, $J_{3ax,3eq}$ = 12.4 Hz, $J_{4,3eq}$ = 4.6 Hz, 2H, 2×H-3eq Neu5Acα); 2.37 (m, 8H, 4×COC*H*$_2$CH$_2$ sp); 2.05 (s, 6H, 2×NCOC*H*$_3$); 1.70 (dd ≈ t, *J* = 12.1 Hz, 2H, 2×H-3_{ax} Neu5Acα); 1.67 (m, 8H, 4×COCH$_2$C*H*$_2$ sp). MS: *m/z*: 1908 $(M)^+$, 1931 $(M+Na)^+$, 1947 $(M+K)^+$.

Neu5Acα-sp1-Gly$_2$-NH(CH$_2$)$_{10}$NH-Gly$_2$-sp1-Neu5Acα (**34**) was prepared according to Protocol 3 from diamine **25**, Neu5Acα-sp1-ONp and NEt_3 in DMSO. Yield: 78%. ^{1}H-NMR (D_2O): 7.45 (m, 8H, 2×*Ar*); 4.75 (d, J_{hem} = 11.1 Hz, 2H, 2×ArC*H*$_2$); 4.56 (d, J_{hem} = 11.0 Hz, 2H, 2×ArC*H*$_2$); 4.06 (s, 4H, 2×C*H*$_2$ Gly); 3.92 (s, 4H, 2×C*H*$_2$ Gly); 3.90 (s, 4H, 2×C*H*$_2$ Gly); 3.86 (dd, $J_{9b,9a}$ = 11.9 Hz, $J_{8,9a}$ = 2.3 Hz, 2H, 2×H-9a Neu5Acα); 3.84 (dd ≈ t, *J* = 10.0 Hz, 2H, 2×H-5 Neu5Acα); 3.76 (ddd, $J_{9a,8}$ = 2.3 Hz, $J_{9b,8}$ = 6.1 Hz, $J_{7,8}$ = 9.1 Hz, 2H, 2×H-8 Neu5Acα); 3.75 (dd, $J_{7,6}$ = 1.4 Hz, $J_{5,6}$ = 10.8 Hz, 2H, 2×H-6 Neu5Acα); 3.70 (ddd, $J_{5,4}$ = 10.0 Hz, $J_{3eq,4}$ = 4.6 Hz, $J_{3ax,4}$ = 11.8 Hz, 2H, 2×H-4 Neu5Acα)*; 3.65* (dd, $J_{8,9b}$ = 6.0 Hz, $J_{9a,9b}$ = 11.9 Hz, 2H, 2×H-9b Neu5Acα); 3.62 (dd, $J_{8,7}$ = 9.0 Hz, $J_{6,7}$ = 1.8 Hz, 2H, 2×H-7 Neu5Acα); 3.18 (m, 4H, 2×CH_2 core); 2.79 (dd, $J_{3ax,3eq}$ = 12.5 Hz, $J_{4,3eq}$ = 4.4 Hz, 2H, 2×H-3eq Neu5Acα); 2.37 (m, 8H, 4×COC*H*$_2$CH$_2$ sp); 2.05 (s, 6H, 2×NCOC*H*$_3$); 1.70 (dd ≈ t, *J* = 12.1 Hz, 2H, 2×H-3_{ax} Neu5Acα); 1.67 (m, 8H, 4×COCH$_2$C*H*$_2$ sp). MS: *m/z*: 1564 $(M)^+$, 1587 $(M+Na)^+$, 1603 $(M+K)^+$.

Neu5Acα-sp1-Gly$_4$-NH(CH$_2$)$_{10}$NH-Gly$_4$-sp1-Neu5Acα (**35**) was prepared according to Protocol 3 from diamine **29**, Neu5Acα-sp1-ONp and NEt_3 in DMSO. Yield: 74% ^{1}H-NMR (D_2O): 7.45 (m, 8H, 2×*Ar*); 4.75 (d, J_{hem} = 11.0 Hz, 2H, 2×ArC*H*$_2$ sp); 4.56 (d, J_{hem} = 11.0 Hz, 2H, 2×ArC*H*$_2$ sp); 4.07 (s, 4H, 2×C*H*$_2$ Gly); 4.03 (s, 4H, 2×C*H*$_2$ Gly);

4.01 (s, 4H, 2×CH_2 Gly); 3.94 (s, 4H, 2×CH_2 Gly); 3.91 (s, 4H, 2×CH_2 Gly); 3.86 (dd, $J_{9b,9a}$ = 11.9 Hz, $J_{8,9a}$ = 2.3 Hz, 2H, 2×H-9a Neu5Acα); 3.84 (dd ≈ t, J = 10.0 Hz, 2H, 2×H-5 Neu5Acα); 3.76 (ddd, $J_{9a,8}$ = 2.3 Hz, $J_{9b,8}$ = 5.9 Hz, $J_{7,8}$ = 9.0 Hz, 2H, 2×H-8 Neu5Acα); 3.75 (dd, $J_{7,6}$ = 1.8 Hz, $J_{5,6}$ = 10.5 Hz, 2H, 2×H-6 Neu5Acα); 3.70 (m, 2H, 2×H-4 Neu5Acα); 3.65 (dd, $J_{8,9b}$ = 5.9 Hz, $J_{9a,9b}$ = 11.9 Hz, 2H, 2×H-9b Neu5Acα); 3.62 (dd, $J_{8,7}$ = 9.0 Hz, $J_{6,7}$ = 1.8 Hz, 2H, 2×H-7 Neu5Acα); 3.18 (m, 4H, 2×NHCH_2 core); 2.79 (dd, $J_{3ax,3eq}$ = 12.5 Hz, $J_{4,3eq}$ = 4.4 Hz, 2H, 2×H-3_{eq} Neu5Acα); 2.36 (m, 8H, 4×COCH_2 sp); 2.06 (s, 6H, 2×NCOCH_3); 1.70 (dd ≈ t, J = 12.1 Hz, 2H, 2×H-3_{ax} Neu5Acα); 1.68 (m, 8H, 2×CH_2CH_2 sp); 1.49–1.40 (m, 4H, 2×CH_2 core), 1.25–1.17 (m, 12H, 6×CH_2 core). MS: *m/z*: 1791 $(M)^+$, 1814 $(M+Na)^+$, 1830 $(M+K)^+$.

Neu5Acα-sp1-Gly$_5$-NH(CH$_2$)$_{10}$NH-Gly$_5$-sp1-Neu5Acα (**36**) was prepared according to Protocol 3 from diamine **31**, Neu5Acα-sp1-ONp and NEt_3 in DMSO. Yield: 53%. ^{1}H-NMR (D_2O): 7.45 (m, 8H, 2×*Ar*); 4.75 (d, J_{hem} = 11.0 Hz, 2H, 2×ArCH_2 sp); 4.56 (d, J_{hem} = 11.0 Hz, 2H, 2×ArCH_2 sp); 4.08 (s, 8H, 4×CH_2 Gly); 4.03 (s, 4H, 2×CH_2 Gly); 4.01 (s, 4H, 2×CH_2 Gly); 3.94 (s, 4H, 2×CH_2 Gly); 3.91 (s, 4H, 2×CH_2 Gly); 3.86 (dd, $J_{9b,9a}$ = 11.9 Hz, $J_{8,9a}$ = 2.3 Hz, 2H, 2×H-9a Neu5Acα); 3.84 (dd ≈ t, J = 10.0 Hz, 2H, 2×H-5 Neu5Acα); 3.76 (ddd, $J_{9a,8}$ = 2.3 Hz, $J_{9b,8}$ = 5.9 Hz, $J_{7,8}$ = 9.0 Hz, 2H, 2×H-8 Neu5Acα); 3.75 (dd, $J_{7,6}$ = 1.8 Hz, $J_{5,6}$ = 10.5 Hz, 2H, 2×H-6 Neu5Acα); 3.70 (m, 2H, 2×H-4 Neu5Acα); 3.65 (dd, $J_{8,9b}$ = 5.9 Hz, $J_{9a,9b}$ = 11.9 Hz, 2H, 2×H-9b Neu5Acα); 3.62 (dd, $J_{8,7}$ = 9.0 Hz, $J_{6,7}$ = 1.8 Hz, 2H, 2×H-7 Neu5Acα); 3.18 (m, 4H, 2×NHCH_2 core); 2.79 (dd, $J_{3ax,3eq}$ = 12.5 Hz, $J_{4,3eq}$ = 4.4 Hz, 2H, 2×H-3_{eq} Neu5Acα); 2.36 (m, 8H, 4×COCH_2 sp); 2.06 (s, 6H, 2×NCOCH_3); 1.70 (dd ≈ t, J = 12.1 Hz, 2H, 2×H-3_{ax}

Neu5Acα);1.70 (m, 8H, 2×CH_2CH_2 sp); 1.49–1.39 (m, 4H, 2×CH_2 core), 1.27–1.20 (m, 12H, 6×CH_2 core). MS: *m/z*: 1906 $(M)^+$, 1929 $(M+Na)^+$, 1945 $(M+K)^+$.

6'SLN-sp2-Gly$_5$-NH(CH_2CH_2O)$_3$$CH_2CH_2$NH-Gly$_5$-sp2-6'SLN (**37**) was prepared according to Protocol 3 from diamine **7**, 6'SLN-sp2-ONp and NEt_3 in DMSO. Yield: 69%. ^{1}H-NMR (D_2O): 4.57 (d, $J_{2,1}$ = 7.1 Hz, 2H, 2×H-1 Gal); 4.47 (d, $J_{2,1}$ = 7.8 Hz, 2H, 2×H-1 GlcNAc); 4.05–3.54 (m, 74H, 2×7H Neu, 2×6H Gal, 2×6H GlcNAc, 2×10H CH_2 Gly, 2×6H CH_2O core, 2×2H OC$H_2$$CH_2CH_2$NH sp); 3.44 (t, 4H, J = 5.2 Hz, NHC$H_2$$CH_2$O core); 3.31–3.26 (m, 2H, 2×NCH sp); 3.24–3.20 (m, 2H, 2×NCH sp); 2.69 (dd, $J_{4,3eq}$ = 4.3 Hz, $J_{3ax,3eq}$ = 12.7 Hz, 2H, 2×H-3$_{eq}$ Neu5Acα); 2.40–2.29 (m, 8H, 4×COCH_2 sp); 2.08 (s, 6H, 2×NCOCH_3); 2.06 (s, 6H, 2×NCOCH_3); 1.80 (m, 4H, 2×OCH_2C$H_2$$CH_2$NH sp); 1.70 (dd ≈ t, J = 12.2 Hz, 2H, 2×H-3$_{ax}$ Neu5Acα); 1.63–1.60 (m, 8H, 2×CH_2CH_2C$H_2$$CH_2$ sp). MS: *m/z*: 2462 $(M)^+$, 2491 $(M+Na)^+$, 2501 $(M+K)^+$.

6'SLN-sp2-Gly$_6$-NH(CH_2CH_2O)$_3$$CH_2CH_2$NH-Gly$_6$-sp2-6'SLN (**38**) was prepared according to Protocol 3 from diamine **9**, 6'SLN-sp2-ONp and NEt_3 in DMSO. Yield: 43%. ^{1}H-NMR (D_2O): 4.57 (d, $J_{2,1}$ = 7.6 Hz, 2H, 2×H-1 Gal); 4.47 (d, $J_{2,1}$ = 7.9 Hz, 2H, 2×H-1 GlcNAc); 4.05–3.54 (m, 78H, 2×7H Neu, 2×6H Gal, 2×6H GlcNAc, 2×12H CH_2 Gly, 6H CH_2O core, 2×2H OC$H_2$$CH_2CH_2$NH sp); 3.44 (t, 4H, J = 5.3 Hz, NHC$H_2$$CH_2$O core); 3.31–3.26 (m, 2H, 2×NCH sp); 3.23–3.18 (m, 2H, 2×NCH sp); 2.69 (dd, $J_{4,3eq}$ = 4.6 Hz, $J_{3ax,3eq}$ = 12.4 Hz, 2H, 2×H-3$_{eq}$ Neu); 2.40–2.29 (m, 8H, 4×COCH_2 sp); 2.08 (s, 6H, 2×NCOCH_3); 2.06 (s, 6H, 2×NCOCH_3); 1.80 (m, 4H, OCH_2C$H_2$$CH_2$NH sp); 1.74 (dd ≈ t, J = 12.2 Hz, 2H, 2×H-3$_{ax}$ Neu); 1.65–1.60 (m, 8H, 2×CH_2CH_2C$H_2$$CH_2$ sp). MS: *m/z*: 2577 $(M)^+$, 2600 $(M+Na)^+$, 2616 $(M+K)^+$.

3'SL-sp3-Gly$_5$-NH(CH$_2$CH$_2$O)$_3$CH$_2$CH$_2$NH-Gly$_5$-sp3-3'SL (**39**) was prepared according to Protocol 3 from diamine **7**, 3'SL-sp3-ONp and NEt_3 in DMSO. Yield: 57%. ^{1}H-NMR (D_2O): 5.05 (d, $J_{2,1}$ = 9.2 Hz, 2H, 2×H-1 Glc); 4.56 (d, $J_{2,1}$ = 7.8 Hz, 2H, 2×H-1 Gal); 4.14 (dd, $J_{2,3}$ = 9.8 Hz, $J_{4,3}$ = 3.3 Hz, 2H, 2×H-3, Gal); 4.05–3.60 (m, 72H; 2×7H Neu, 2×5H Gal, 2×6H Glc, 2×12H CH_2 Gly, 2×6H CH_2O core); 3.49 (m, 2H, 2×H-2 Glc); 3.45 (t, J = 5.3 Hz, 4H, 2×NCH_2 core); 2.79 (dd, $J_{4,3eq}$ = 4.5 Hz, $J_{3ax,3eq}$ = 12.2 Hz, 2H, 2×H-3_{eq} Neu); 2.39 (m, 8H, 4×COCH_2 sp); 2.06 (s, 6H, 2×NCOCH_3); 1.82 (dd ≈ t, J = 12.2 Hz, 2H, 2×H-3_{ax} Neu); 1.69–1.64 (m, 8H, 2×CH_2CH_2C$H_2$$CH_2$ sp). MS: *m/z*: 2378 $(M)^+$, 2401 $(M+Na)^+$, 2417 $(M+K)^+$.

3'SL-sp3-Gly$_6$-NH(CH$_2$CH$_2$O)$_3$CH$_2$CH$_2$NH-Gly$_6$-sp3-3'SL (**40**) was prepared according to Protocol 3 from diamine **9**, 3'SL-sp3-ONp and NEt_3 in DMSO. Yield: 51%. ^{1}H-NMR (D_2O): 5.05 (d, $J_{2,1}$ = 9.2 Hz, 2H, 2×H-1 Glc); 4.57 (d, $J_{2,1}$ = 7.9 Hz, 2H, 2×H-1 Gal); 4.14 (dd, $J_{2,3}$ = 9.9 Hz, $J_{4,3}$ = 3.2 Hz, 2H, 2×H-3, Gal); 4.03–3.59 (m, 76H; 2×7H Neu, 2×5H Gal, 2×6H Glc, 2×14H CH_2 Gly, 2×6H CH_2O core); 3.49 (m, 2H, 2×H-2 Glc); 3.45 (t, J = 5.3 Hz, 4H, 2×NCH_2 core); 2.79 (dd, $J_{4,3eq}$ = 4.6 Hz, $J_{3ax,3eq}$ = 12.4 Hz, 2H, 2×H-3_{eq} Neu); 2.39 (m, 8H, 4×COCH_2 sp); 2.06 (s, 6H, 2×NHCOCH_3); 1.82 (dd ≈ t, J = 12.2 Hz, 2H, 2×H-3_{ax} Neu); 1.69–1.64 (m, 8H, 2×CH_2CH_2C$H_2$$CH_2$ sp). MS: *m/z*: 2492 $(M)^+$, 2515 $(M+Na)^+$, 2531 $(M+K)^+$.

Dynamic light scattering experiments

Light scattering of aqueous solutions of biantennary oligoglycines was studied with an analyzer of submicron particle size "Malvern HPPS" (UK). After preparation of aqueous (Milli-Q) solutions of oigoglycine salts in concentration 0.01–0.1 mg/mL

instrument reading was recorded (t = 0, pH < 5). After this 1–2 equiv of base (0.1 M aqueous solution of $NaHCO_3$ or Na_2CO_3) per amino group was added to the solution of the analyzed oligoglycine salt (t > 0, pH 6–8) and recorded instrument readings in fixed periods of time. In case of formation of large associates (intense opalescence, sedimentation) with the dimensions exceeding the working limit of the instrument, the experiment was stopped.

For experiments with biantennary sialooligoglycines their aqueous (Milli-Q) solutions with concentration 0.1 mg/mL were used.

Scanning force microscopy (SFM)

The samples were imaged with Nanoscope IIIa instrument (Digital Instruments, USA) with commercial silicon nitride cantilevers with 0.06, 0.12, and 0.32 Nm^{-1} force constants were used for the measurements in contact mode in liquid cell. Cantilevers with resonance frequency about 300 kHz and force constant 42 Nm^{-1} were used for SFM tapping mode in air. Software WSxM (Nanotec Electronica, Spain) was used for image treatment. Purified water (Fluka) was used for preparation of solutions.

Scanning in air: 1–2 equiv of 0.1 M of aqueous solution $NaHCO_3$ or Na_2CO_3 per amino group (pH ~ 6–8) was added for deprotonation to a freshly prepared solution of oligoglycine salt (0.1 or 1.0 mg/mL; pH < 5), and incubated for a settled time period in the range 0–90 min. Then the solution was applied on the freshly cleaved mica or graphite, and kept for the settled period of time within the range 0–10 min. Liquid was

removed from the surface by spin coating or in nitrogen flow. Structures formed on the surface were visualized in tapping mode SFM.

Scanning in liquid cell: A plate of freshly cleaved mica (1×1 cm^2) was placed in liquid cell. The cell was filled with water (25 μl) and the instrument was set up. Then water was changed with freshly prepared solution of deprotonated peptide (see Scanning in air above) and the surface was scanned in contact mode SFM in fixed time periods.

Influenza virus receptor-binding inhibition assay was done completely as described in [4].

References

1. Gershkovich, A.A., Kibirev, V.Ch. *Chemical synthesis of peptides*. Naukova Dumka: Kiev, Ukraine, 1992.
2. Katritzky, A.R.; Singh, S.K.; Meher, N.K.; Doskocz, J.; Suzuki, K.; Jiang, R.; Sommen, G.L.; Ciaramitaro, D.A.; Steel, P.J. *ARKIVOC* **2006**, *V*, 43–62.
3. Katritzky, A.R.; Meher, N.K.; Hanci, S.; Gyanda, R.; Tala, S.R.; Mathai, S.; Duran, R.S.; Bernard, S.; Sabri, F.; Singh, S.K.; Doskocz, J.; Ciaramitar, D.A. *Journal of Polymer Science Part A: Polymer Chemistry* **2008**, *46*, 238–256.
4. Gambaryan, A.S.; Matrosovich M.N. *J. Virol. Methods* **1992**, *39,* 11–123.
